# Computational Study of Complexation in LiH:nNH_3_ (*n* = 1–4) Clusters: An Interplay Among Hydrogen, Dihydrogen, and Lithium Bonds

**DOI:** 10.1002/jcc.70114

**Published:** 2025-04-19

**Authors:** Krishna, Lalit Kumar Saini, Mukesh Pandey

**Affiliations:** ^1^ Department of Physics Sardar Vallabhbhai National Institute of Technology Surat India; ^2^ Atomic and Molecular Physics Division Bhabha Atomic Research Centre Mumbai India

**Keywords:** Complexation, Many‐body energy decomposition analysis, Molecular clusters, Semi‐empirical quantum chemistry methods

## Abstract

Ab initio and density functional theory (DFT) calculations are employed to investigate LiH:nNH_3_ (*n* = 1–4) cluster complexes. The nature of the interactions is analyzed using molecular electrostatic potential maps, quantum theory of atoms in molecules, delocalization indices, and electron density difference maps. In the presence of LiH, NH_3_ molecules engage in several types of noncovalent interactions, namely, hydrogen bonding (HB), lithium bonding (LB), and dihydrogen bonding (DHB). The LiH:NH_3_ dimer is stabilized primarily through Li···N interactions. The role of these noncovalent interactions in complexes having more than one NH_3_ molecule, for example, hetero‐trimer, tetramer, and pentamer structures, is also examined. Increasing the number of NH_3_ molecules enhances the number of HB sites. Additionally, the strengths of LB and DHB interactions associated with HB‐bonded NH_3_ molecules increase. Interaction energy estimates and many‐body energy decomposition analysis suggest that increasing NH_3_ molecules increases cooperativity, approaching ~10% of the total interaction's energy in the case of tetramers and pentamers.

## Graphical abstract

Complexation in aggregates of up to four NH_3_ molecules with LiH (LiH:nNH_3_) has been proposed. We employ quantum chemical treatment using MEP, QTAIM, electron density difference maps, and many‐body energy decomposition analysis. The role of hydrogen bonding (HB), lithium bonding (LB), and dihydrogen bonding (DHB) has been observed in this complexation. A cooperative interplay has been observed among HB, DHB, and LB. This study finds implications in NH_3_‐based hydrogen generation and hydrogen storage systems.

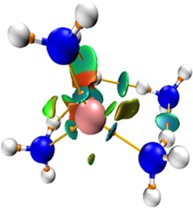

## 1 Introduction

The intermolecular interactions are fundamental to understanding condensed phases and aggregation phenomena. The noncovalent bonds, much weaker than the ionic, covalent, or metallic bonds, govern the forces between the various segments in supra‐molecules, molecular aggregates, and clusters, etc., that are not directly connected by covalent bonds [1, 2]. They are of major importance, as they are involved in the supra‐molecular structure of macromolecules like proteins [3]. Among various noncovalent interactions, the hydrogen bond is the most thoroughly investigated [4, 5, 6, 7, 8, 9, 10]. Recently, other kinds of noncovalent interactions, such as lithium bonding (LB) [11, 12, 13] and dihydrogen bonding (DHB) [14, 15], have also garnered attention. LB (A–Li···B) was theoretically described by Kollman et al. in 1970 and experimentally confirmed by Ault and Pimentel in 1975 using matrix isolation infrared spectroscopy [12]. Although studies on three‐center lithium bonds (LiBs) are relatively rare, still they have their own importance in chemical bonding, as reported in several studies [11, 12, 13, 16, 17, 18]; specifically, the recognition of Li‐bonding as an important type of three‐center interaction has also been discussed by Sannigrahi et al. [17] in various Li‐dimer systems, followed by ^7^Li‐NMR studies on Mo_2_LiH_2_, Mo_2_Li_2_H_4_, and Mo_6_Li_9_H_18_ Clusters by Marina et al. [16], revealing the formation of Mo–H–Li three‐center–two‐electron bonds, supplemented by the coordination of the Mo≡Mo bond to the Li^+^ ion. Another class of interaction to be explored is the DHB, which is strongly influenced by HBs. The mutual reinforcement of these interactions, characterized by their non‐additive nature, leads to the so‐called “cooperativity phenomenon” [19, 20, 21], making their interplay another important area to be explored. Although the contribution of non‐additive components is relatively small, they are critical in stabilizing the structures of complex systems, as observed in many systems including water clusters [21], focusing on cooperativity effects in systems featuring HB and LB [22, 23, 24], as well as their influence on other interactions, such as halogen bonding (XB) [25, 26]. Similarly, the interaction between HBs and DHBs has also been closely examined [27, 28]. This study aims to investigate the interplay between lithium, hydrogen, and DHB in the LiH:nNH_3_ (*n* = 1–4) clusters and their associated cooperativity effects. Also, this study finds importance, as LiH–NH_3_ complexes are known for their importance in hydrogen energy production and storage [29, 30, 31, 32].

The role of NH_3_ molecule functioning both as proton donors as well as proton acceptors and stabilizing the NH_3_ clusters through hydrogen bonding (X–H⋯Y) is well known [33, 34, 35]. This study discusses the complexation of NH_3_ molecules with lithium hydride (LiH), an ionic molecule (Li^+^ H^−^), introducing noncovalent interactions in terms of DHBs (M–H^−^⋯H^+^–Y) and LBs (H–Li⋯N). Herein, the cooperative behavior among the various noncovalent interactions is corroborated with quantum chemical approaches, like quantum theory of atoms in molecules (QTAIM), molecular electrostatic potential (MEP) maps, and electron density difference (EDD) maps. The interplay among the various noncovalent interactions stabilizes the different geometrical structures. The role of additive as well as non‐additive contributions of different energy components toward the stability and instability of these complexes is examined using many‐body interaction energies combined with energy decomposition analysis (EDA). These findings provide insights into the fundamental mechanisms of complexation in LiH:nNH_3_ clusters and the intricate role of noncovalent forces.

## 2 Theoretical and Computational

This study begins by thoroughly examining the potential energy surfaces (PESs) of LiH:nNH_3_ (*n* = 1–4) clusters in order to adequately understand the nature and strength of noncovalent interactions. This exploration was conducted by initially predicting the global as well as local minimum energy structures on these PESs using the ABCluster code [36, 37] for global optimization of cluster geometries. Notably, ABCluster offers a reasonable balance between accuracy and computational efficiency, making it a reliable tool for generating initial structures for further optimization at a higher level of theory [38, 39, 40, 41]. ABCluster performs a global optimization on given PESs and locates the global and local minima using only electrostatics and Lennard‐Jones interactions. The potential energy (U) of the rigid molecular cluster used in ABCluster is assumed to be of the following form:

(1) $$\begin{array}{c} U_{\text{CHARMM}}=\sum_{I=1}^{N}\;\sum_{I<J}^{N}\;\sum_{i_{I}\in I}\;\sum_{j_{J}\in J}\\ \;\left(\frac{e^{2}}{4\pi \varepsilon_{0}}\frac{q_{i_{I}}q_{j_{J}}}{r_{i_{I}j_{J}}}+4\pi \epsilon_{i_{I}j_{J}}\left({\left(\frac{\sigma_{i_{I}cj_{J}}}{r_{i_{I}j_{J}}}\right)}^{12}-{\left(\frac{\sigma_{i_{I}j_{J}}}{r_{i_{I}j_{J}}}\right)}^{6}\right)\right)\\ \;+\sum_{I=1}^{N}\;\sum_{i_{I}\in I}\left(q_{i_{I}}\mathrm{eF}z_{i_{I}}\right) \end{array}$$

where I and J are the indices of the molecules, $i_{I}$ and $j_{J}$ are the indices of the atoms in molecules I and J, respectively, and $r_{i_{I}j_{J}}$ is the distance between atom $i_{I}$ and $j_{J}$. The first term describes the intermolecular Coulomb and Lennard‐Jones interactions, where Lennard‐Jones well depth *ϵ* and width *σ* are the CHARMM parameters. The second term describes the interactions between molecules in the external static electric field for a molecule which is zero in this case. Three parameters need to be set in the ABCluster program: the scout limit *g*_*limit*_, the size of the population of trial solutions SN and the maximum cycle number *g*_*max*_. In the present investigations, these parameters were set as *g*_*limit*_ = 4; SN = 60 and *g*_*max*_ = 500. The initial geometry of lithium‐hydride ammonia complexes (LiH:nNH_3_; *n* = 1–4) were generated using ABCluster and then fully optimized at the RI‐MP2 “No frozen core” level of theory in conjunction with 6‐311++g(2df,2pd) and aug‐cc‐pVTZ basis set. Choice of these basis sets is fairly good, as Bene et al. has also successfully employed them in ab initio molecular dynamics studies of bimolecular complex formation of NH_3_ in various hydrides [42], for computing the stabilization energies. In all cases, vibrational frequencies were calculated in order to confirm that these structures correspond to true minima and to obtain the zero‐point vibrational. All calculations were carried out with the ORCA [43] program package (v5.0.4) with highest convergence criteria pertaining to SCF calculations and very tight optimization parameters. Interaction energies of LiH:NH_3_ hetero‐dimer were computed using DLPNO‐CCSD(T)/aug‐cc‐pVTZ [44] under TightPNO convergence criteria and RI‐MP2, B3LYP‐D3(BJ)/aug‐cc‐pVDZ/6‐311++g(2df,2pd)/aug‐cc‐pVTZ as the difference in energy between the complex, and the sum of the energies of the monomers, using the monomer geometries from the optimized complex. Interaction energies were corrected by the counterpoise procedure [45]. In addition, to calculate the interaction energy and to study the effect of non‐additive term, that is, cooperative and anti‐cooperative effects in molecular cluster systems, we have used many‐body energy decomposition analysis (MB‐EDA) [46] method at B3LYP‐D3(BJ)/aug‐cc‐pVDZ, where, the EDA scheme is combined with many‐body expansion (MBE). In cluster systems, the interactions between two molecules are influenced by their surrounding ones. The MBE method provides an effective approach for studying the many‐body effects of cluster systems. The MBE for a cluster system of *N* monomers is given by

(2) $$\begin{array}{c} E=\sum_{I=1}^{N}E_{I}^{\left(1\right)}+\frac{1}{2!}\sum_{I_{1}\neq I_{2}}^{N}{∆E}_{I_{1}I_{2}}^{\left(2\right)}\\ \;+\ldots +\frac{1}{n!}\sum_{I_{1}\neq \ldots \neq I_{n}}^{N}{∆E}_{I_{1\ldots I_{n}}}^{\left(n\right)}+{∆E}_{I_{1\ldots I_{N}}}^{\left(N\right)} \end{array}$$

where *E* is the energy of the whole cluster system, and the superscript number n in parentheses denotes the n‐body energy and *I* denote individual monomers. The first term refers to one‐body energy term (Equation (2)) indicating the sum of all individual monomer's energy; the second term refers to the two‐body energy term indicating the pairwise interaction energy, similarly the *n*th‐body energy term (n≥3) indicates combination of such n energy terms, and results in the cooperative or anti‐cooperative interaction energy of n monomers. The subtraction of one body energy from total energy gives the interaction energy

(3) $$\begin{array}{c} \begin{array}{c} ∆E^{\mathrm{int}}\equiv E-\sum_{I=1}^{N}E_{I}^{\left(1\right)}=\frac{1}{2!}\sum_{I_{1}\neq I_{2}}^{N}{∆E}_{I_{1}I_{2}}^{\left(2\right)}\\ \;+\ldots +\frac{1}{n!}\sum_{I_{1}\neq \ldots \neq I_{n}}^{N}{∆E}_{I_{1}\ldots I_{n}}^{\left(n\right)}+\ldots +{∆E}_{I_{1}\ldots I_{N}}^{\left(N\right)} \end{array} \end{array}$$

Thus, the one‐, two‐, and three‐body energies are represented as follows:

(4) $$\begin{array}{c} E_{I}^{\left(1\right)}=E_{I}\; \end{array}$$

(5) $$\begin{array}{c} ∆E_{\mathrm{ij}}^{\left(2\right)}=E_{\mathrm{ij}}-E_{i}^{\left(1\right)}-E_{j}^{\left(1\right)}\; \end{array}$$

(6) $$\begin{array}{c} ∆E_{\mathrm{ijk}}^{\left(3\right)}=E_{\mathrm{ijk}}-E_{i}^{\left(1\right)}-E_{j}^{\left(1\right)}-E_{k}^{\left(1\right)}-∆E_{\mathrm{ij}}^{\left(2\right)}-∆E_{\mathrm{ik}}^{\left(2\right)}-∆E_{\mathrm{jk}}^{\left(2\right)}\; \end{array}$$

This MBE scheme is combined with EDA, based on target state optimization (TSO)‐SCF calculations, as described by Zhang et al. [47]. The total intermolecular interaction energy is decomposed into five chemically meaningful terms, namely, electrostatic ($∆E^{\mathrm{el}}$), exchange–correlation ($∆E^{\mathrm{xc}}$), polarization, ($∆E^{\mathrm{pl}}$), charge transfer ($∆E^{\mathrm{ct}}$), and dispersion ($∆E^{\text{disp}}$) terms, that is,

(7) $$∆E^{\mathrm{int}}=∆E^{\mathrm{el}}+∆E^{\mathrm{xc}}+\;∆E^{\mathrm{pl}}+∆E^{\mathrm{ct}}+∆E^{\text{disp}}$$

The electrostatic term represents the semiclassical Coulombic interaction of charged particles from different monomers, the second term in Equation (7) contains electronic exchange. This denotes the difference in exchange–correlation energy of the super‐molecule (with the combination wave function) and the sum of exchange–correlation energy of monomers (with their individual optimized wave function). Generally, $∆E^{\mathrm{xc}}$ has a positive value which manifests as a steric hindrance effect. In the macroscopic view, the polarization energy ($∆E^{\mathrm{pl}}$) term is calculated as a difference between the energy of the variationally optimized charge‐localized state and charge‐localized diabatic state. The fourth term in Equation (7) contain charge transfer. This is the charge transfer energy that results from electron transfer from the occupied MOs of one monomer into the virtual MOs of its neighbouring monomer. Finally, the dispersion term ($∆E^{\text{disp}}$) is the Grimme's D3(BJ) dispersion [48], which is calculated as dispersion energy difference between the super‐molecule and the sum of its monomers.

QTAIM [49] is employed for understanding molecular interactions and the molecular topology. It is a highly effective method for analyzing molecular interactions based on the electron density at critical points (CPs). Software like Multiwfn, VMD, and Avogadro were utilized for analytical and visualization purposes [50, 51, 52].

## 3 Results and Discussion

The complexation of LiH with ammonia molecules is described below. In this context, the description of MEP maps of the monomers, namely, LiH, NH_3_, and the complex LiH:NH_3_, plays an instrumental role in different geometrical structures. We first describe the complexation mechanism in LiH:NH_3_ using the MEP maps. Subsequently, the complexation mechanisms in hetero‐dimer (LiH:NH_3_) and hetero‐trimer (LiH:2NH_3_) using the results based on QTAIM, delocalization index, EDD map, and noncovalent interaction index (NCI) are described. This understanding is further extended to higher order complexes, that is, hetero‐tetramer (LiH:3NH_3_) and hetero‐pentamer (LiH:4NH_3_). Finally, the interaction energy is estimated in terms of many‐body components, and the decomposition of the interaction energy into five chemically meaningful energy components is performed to get further insight into this complexation.

### 3.1 Complexation Mechanism in Different Geometrical Conformers of LiH:nNH_3_ (*n* = 1–4) Clusters

The PES of the LiH:NH_3_ heterodimer contains a single minimum while the number of minima increases as the number of NH_3_ molecules increases. ABCluster generates numerous such minima, but after optimization at a higher level of theory, all these minima converge to only a few distinct minima, both in terms of their energy and structure. Thus, all these converged local minima are considered in addition to the global minima. DFT and MP2 calculations using different basis sets viz., 6‐311++g(2df,2pd), aug‐cc‐pVDZ, aug‐cc‐pVTZ, were performed to corroborate that the number of local minima in the LiH:nNH_3_ system is independent of the computational level of theory used. Moreover, the total number of local minima and their energetic ordering remain unchanged with the different basis sets used.

In LiH:NH_3_ complex, Li interacts directly with the N atom, as evident from the global minima, converged with RI‐MP2/6‐311++G(2df,2pd) level of theory. Indeed, it needs to be mentioned that among the various local minima, the local minima corresponding to LiH···NH_3_ heterodimer, comprising interatomic distances, namely, LiH···HNH_2_ (with H···H ~ 1.843 Å, a condition favorable for DHB) and LiH···NH_3_ (with H···N ~ 1.863 Å, a condition favorable for hydrogen bonding) also finally converged into a geometry similar to A1. The initial geometry of these local minima along with their optimized geometries with RI‐MP2/6‐311++G(2df,2pd) are given in the supplementary, as Figure S1. This is perhaps because, in LiH, the large electronegativity difference makes Li highly electropositive and a strong Lewis acid, favoring coordination with the lone pair of nitrogen over H···H interactions. Likewise, the Lewis‐basic nitrogen in NH_3_ also preferentially binds to Li. However, in all other complexes (LiH:nNH_3_; *n* = 2–4), the various kinds of interactions, that is, N···H, H···H, and N···Li do also appear, resulting in more than one conformer (to be discussed later).

**FIGURE 1:**
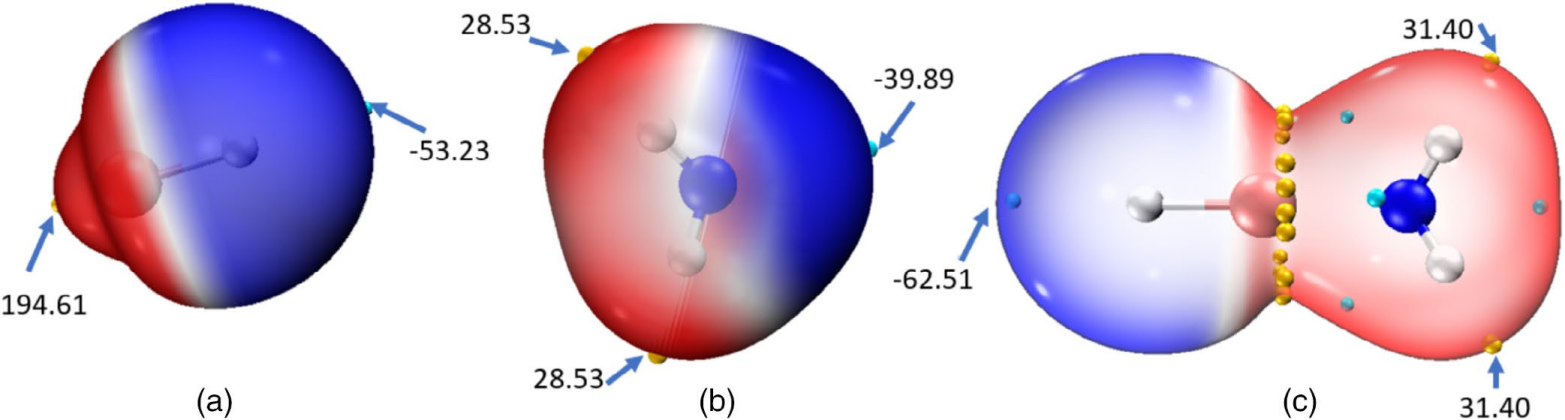
Molecular electrostatic potential calculated at RI‐MP2/6‐311++g(2df,2pd): (a) LiH (left), (b) NH_3_ (middle), and (c) LiH–NH_3_ (right). The cyan and yellow dots denote surface minima and maxima, and red and blue colors represent positive and negative electrostatic potentials, respectively. Isosurface 0.001 a.u.

Next, the distribution of electrostatic potentials on molecular geometries of LiH, NH_3_, and the global minimum structure of LiH:NH_3_, evident from the MEP maps (Figure 1), describes how the molecule's positive region aligns with its negative region. The MEP maps corresponding to each of the monomers are illustrated in Figure 1, where red and blue regions correspond to positive and negative potentials, respectively. As shown by the isosurface plots in Figure 1a, the maximum value of the LiH potential (194.61 kcal‐mol^−1^) on the van der Waals (vdW) surface lies above the Li atom, while the minimum value (−53.23 kcal‐mol^−1^) lies at the H atom. It may be noted that in the case of NH_3_, the potential around the Hydrogen and Nitrogen is largely positive and negative, respectively. The complex, that is, LiH:NH_3_ (Figure 1c) shows the most significant change in the MEP map, that is, the hydric hydrogen becomes more negative, while the protonic hydrogen becomes more positive. Herein, the positive ring appearing across the neck around the Li atom attracts more ammonia molecules via lithium bonds. It is this Li bond that plays an important role in the complexation mechanism of LiH with a greater number of NH_3_ molecules. Although polarization effects are present due to the polarity of LiH and NH_3_, electrostatic interactions still remain the dominant stabilizing factor, outweighing the effects of polarization in these complexes. This kind of observation is also reported by Sannigrahi et al. [17] in their studies of Li‐bonded complexes, where the stability of these complexes stems mainly from the electrostatic interaction with some polarization effects.

**FIGURE 2:**
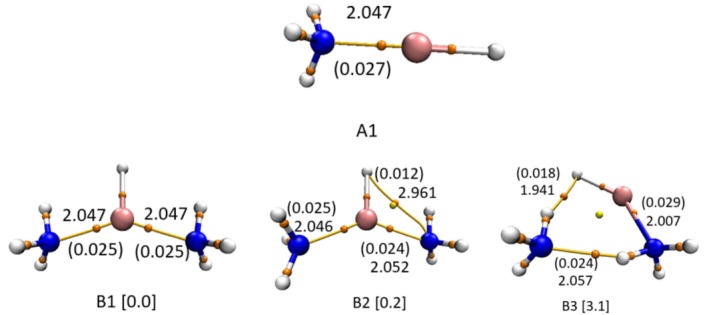
Molecular graph of different geometrical conformers corresponding to hetero‐dimer (LiH:NH_3_) (A1) and hetero‐trimer (LiH:2NH_3_) (B1, B2, and B3) optimized at the RI‐MP2/6‐311++G(2df,2pd) computational level. Solid yellow lines represent bond paths with orange spheres (small) representing BCPs along with their respective interatomic distances in Å; the yellow circle represents RCPs. Electron density (a.u.) at the respective BCPs is given in parentheses. Blue, purple, and silver colors represent nitrogen, lithium, and hydrogen, respectively. Values in square brackets are the relative energy (in kcal‐mol^−1^) with respect to their most stable conformer optimized at B3LYP‐D3(BJ)/aug‐cc‐pVDZ.

The different minimum energy structures of lithium‐hydride ammonia complexes for hetero‐dimers (LiH:NH_3_) and hetero‐trimers (LiH:2NH_3_) along with their respective molecular graphs optimized at RI‐MP2/6‐311++G(2df,2pd) are shown in Figure 2. The corresponding geometrical parameters, obtained at the different levels of theory, are summarized in Table S1. The LiH:NH_3_ hetero‐dimer features only a single minimum, while the LiH:2NH_3_ hetero‐trimer exhibits three distinct minima: B1, B2, and B3. The conformers B1 and B2 exhibit direct bonding of the Li atom with both the NH_3_ molecules in the trans‐position; the B3 conformer shows a cyclic structure. All three minima are stabilized by Li···N interactions. The specific bond paths for each of the conformers are also shown in Figure 2. This bonding pattern, as supported by QTAIM analysis (via the electron density at bond critical point (BCP), as shown in Table S2), provides important information about the noncovalent interactions. In the case of A1, the distance between the Li···N is 2.047 Å and the electron density ρ and Laplacian of electron density ∇^2^ρ at the BCP are 0.027 a.u. and 0.163 a.u., respectively. For B1, this distance that is, R(Li···N) is still 2.047 Å, but the electron density decreases to 0.025 a.u. However, B2 exhibits two distinct values of R(Li···N) that is, 2.046 Å, 2.052 Å, and one value for the N···H bond path with interatomic distance ~2.961 Å. The N···H noncovalent bond involving the hydride atom is quite unusual and is therefore examined critically. The values of ρ and ∇^2^ρ at the N···H BCP are 0.012 a.u. and 0.0257 a.u., respectively. Although these values follow the characteristics of noncovalent bonding, they do not qualify as hydrogen bonding, as this interaction does not follow the basic criterion of hydrogen bonding (X‐H···Y) [53]. Instead, this appears to result from topological proximity rather than bonding, with factors such as nuclear vibrations and non‐nuclear attractors potentially influencing the observed bond path. Various groups have also observed that QTAIM analysis sometimes predicts bond paths between atoms, which traditional chemical intuition does not qualify as a chemical bond [54, 55]. It is therefore believed that such a bond path arises due to the topological proximity rather than the bonding.

**FIGURE 3:**
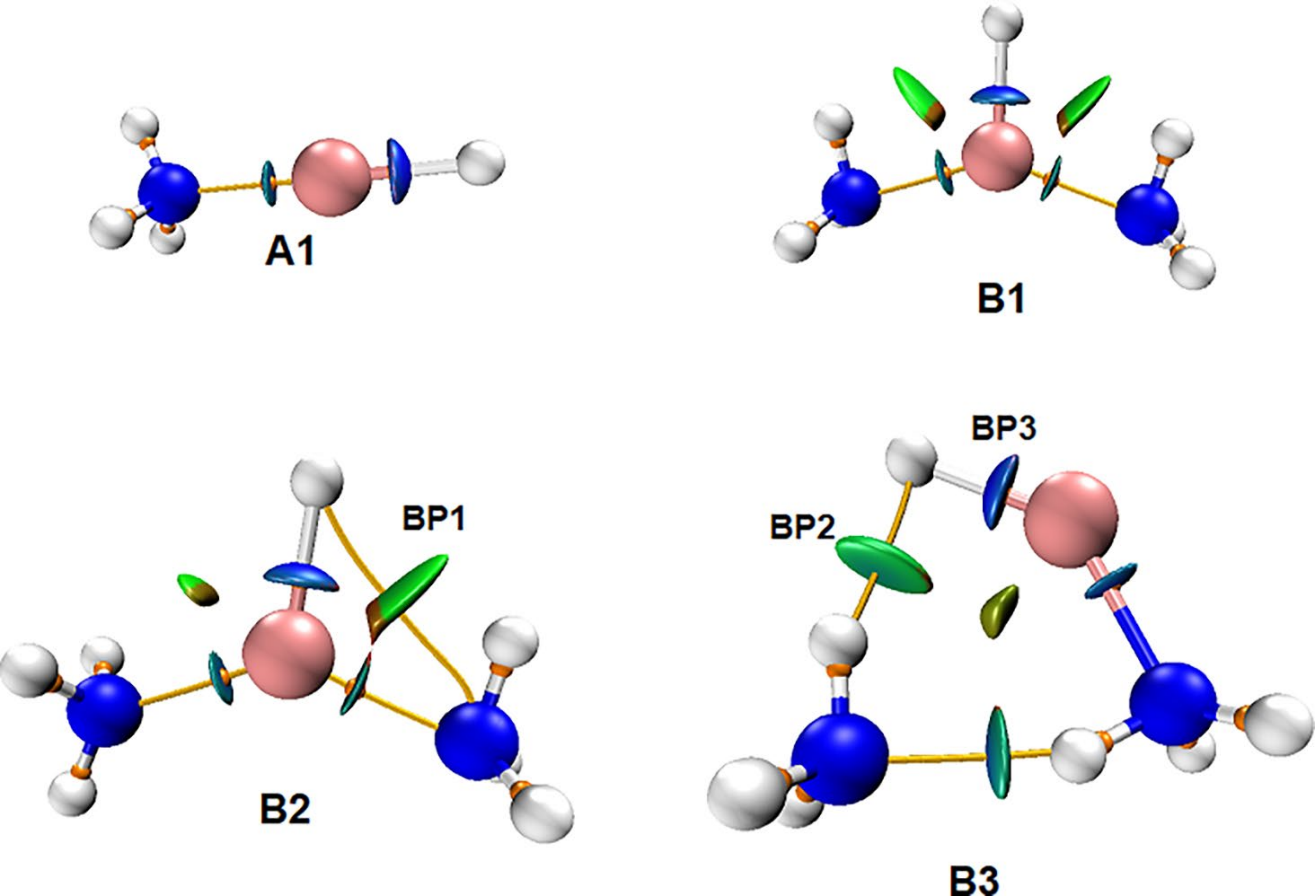
NCI plot of various conformers of LiH:nNH_3_ (*n* = 1–2); blue and cyan discs show strong attractive interaction, green discs show weak attractive, and light green semi‐discs are for the weak van der Waals interaction. BP1 depicts van der Waals interaction in B2 conformer, while BP2 and BP3 are the bond paths representing dihydrogen and hydrogen bonding, respectively, in B3 conformer.

In order to further confirm the topological proximity, estimation of delocalization index (DI), which is the measure of the electron sharing between two atoms, has been carried out. It has been found in our calculations that except for this specific bond path (in B2 conformer), the DI values for N···H interaction have a range from 0.046 to 0.092 (as estimated for other different conformers, including tetramers and pentamers). However, for the B2 conformer, this value is 0.026, which is significantly lower. Further, since the DI value also depends on the polarity of the proton‐donor molecule [56], for Li···N interactions, these DI values are 0.451 and 0.443 for the shorter and longer R(Li···N) distances, respectively. On the contrary, DI values for B3, B1, and A1 are 0.466, 0.444, and 0.447, respectively. To gain further insights into the issue of topological proximity, NCI plots [57] were constructed for different conformers of LiH:nNH_3_ (*n* = 1–2) complex (Figure 3). The NCI plot of the B2 conformer suggests a vdW kind of interaction between Li‐H···N atoms, as depicted by BP1. In comparison, the B3 conformer exhibits additional H···H (DHB) and N···H interactions (HB), shown by BP2 and BP3, respectively, in Figure 3. For the B3 conformer, the R(Li···N) distance is 0.04 Å shorter and the corresponding ρ is 0.004 a.u. higher in comparison with B1. The values of ρ and ∇^2^ρ for H···H (DHB) are 0.018 a.u. and 0.032 a.u., while for N···H (HB) interactions, these values are 0.024 a.u. and 0.066 a.u., respectively. Structural analysis of the B3 structure reveals that ammonia molecules which participate in (H···H) interaction act as a proton acceptor as well as a proton donor. Additionally, the ammonia molecule that donates a proton to form the hydrogen bond receives electron density, which in turn strengthens the Li···N interaction, and this arrangement enables the formation of an H···H bond path. This kind of redistribution in electron density suggests a cooperative interplay between lithium, hydrogen, and dihydrogen bonds.

**FIGURE 4:**
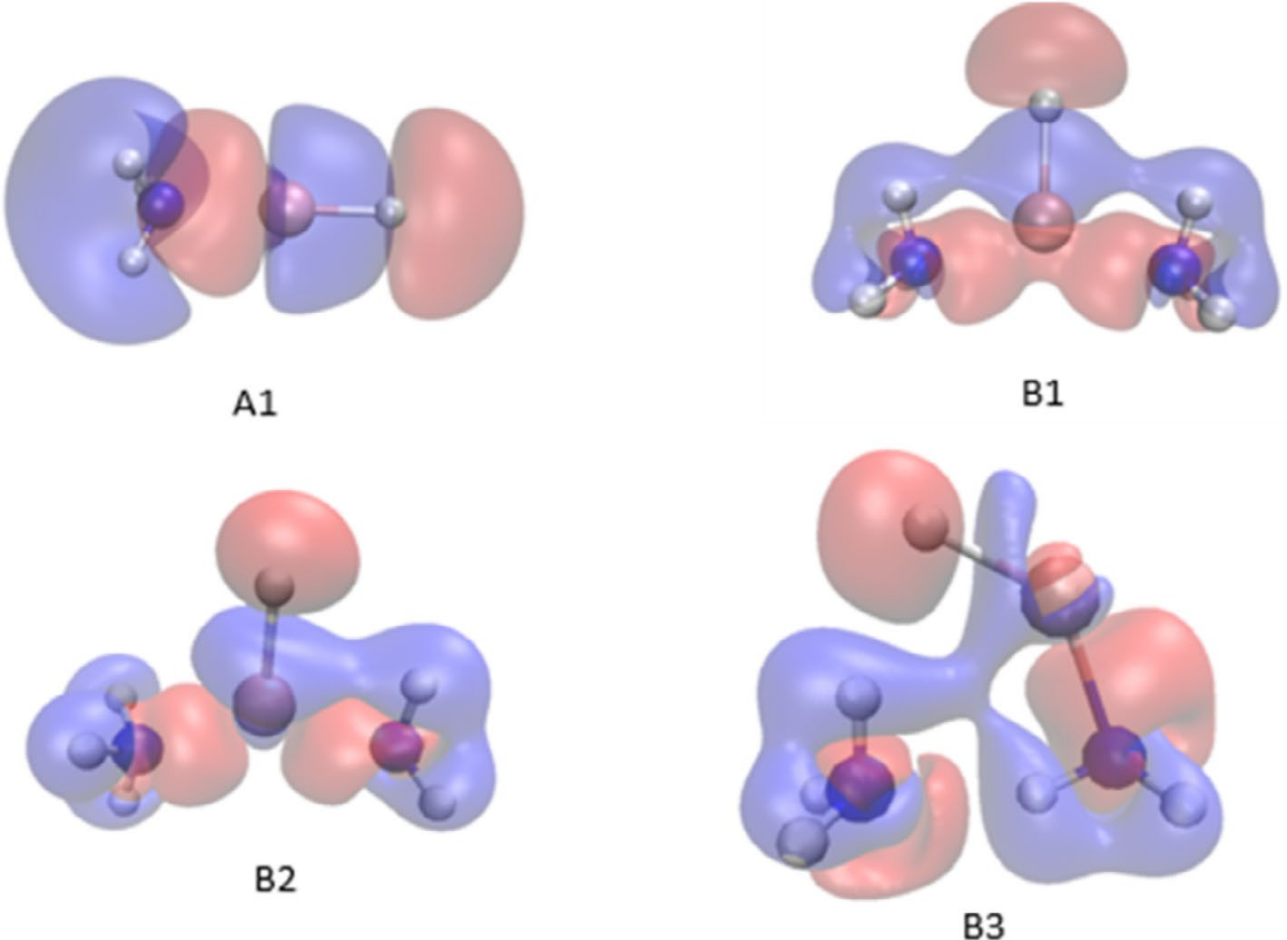
Electron density shifts occurring in the LiH:nNH_3_ (*n* = 1–2) complexes optimized at the RI‐MP2/6‐311++G(2df,2pd) level of theory. Red and blue regions refer to the gain and loss of density in complexes, respectively, relative to isolated monomers. Isosurface value 0.001 a.u.

As the two‐molecule complex begin to interact with one another, they perturb one another's electron clouds. Redistribution of total electron density occur as a result of the complexation. EDD of these complexes are performed, as shown in Figure 4. EDD is defined as $\rho =\rho_{\text{complex}}-\sum \rho_{\text{molecule}}$ where, $\rho_{\text{complex}}$ is the electron density of complex and $\rho_{\text{molecule}}$ is the electron density of each molecule in the complex. The EDD map for LiH:nNH_3_ (*n* = 1 and 2) showing red and blue lines, indicate gain and loss of electron density respectively, relative to the isolated monomers. The H–Li···N–H_3_ lithium bond in each complex is confirmed by a characteristic loss of electron density around the hydrogen atom (belonging to an ammonia molecule) and in the vicinity of lone pair region of the nitrogen atom; and an increase in electron density around the lithium atom. Analyzing EDD of B3 revel that electron density is accumulated around N of ammonia molecule (which act as a proton donor) as well as around Li···N bond. This further corroborates the effect of strengthening of lithium bond and formation of hydrogen and dihydrogen bonds. For qualitative analysis of charge transfer we evaluated the AIM charge on the LiH:nNH_3_ complexes, as given in Table S3. It is observed that in these complexes NH_3_ molecules get more polarized, since nitrogen being more negatively charged and hydrogen being more positively charged compared to their non‐complexation state. Hydrogen atom (on NH_3_) taking part both in hydrogen and DHB in a LiH:nNH_3_ complex, is therefore now more positively charged compared to that without bonding.

**FIGURE 5:**
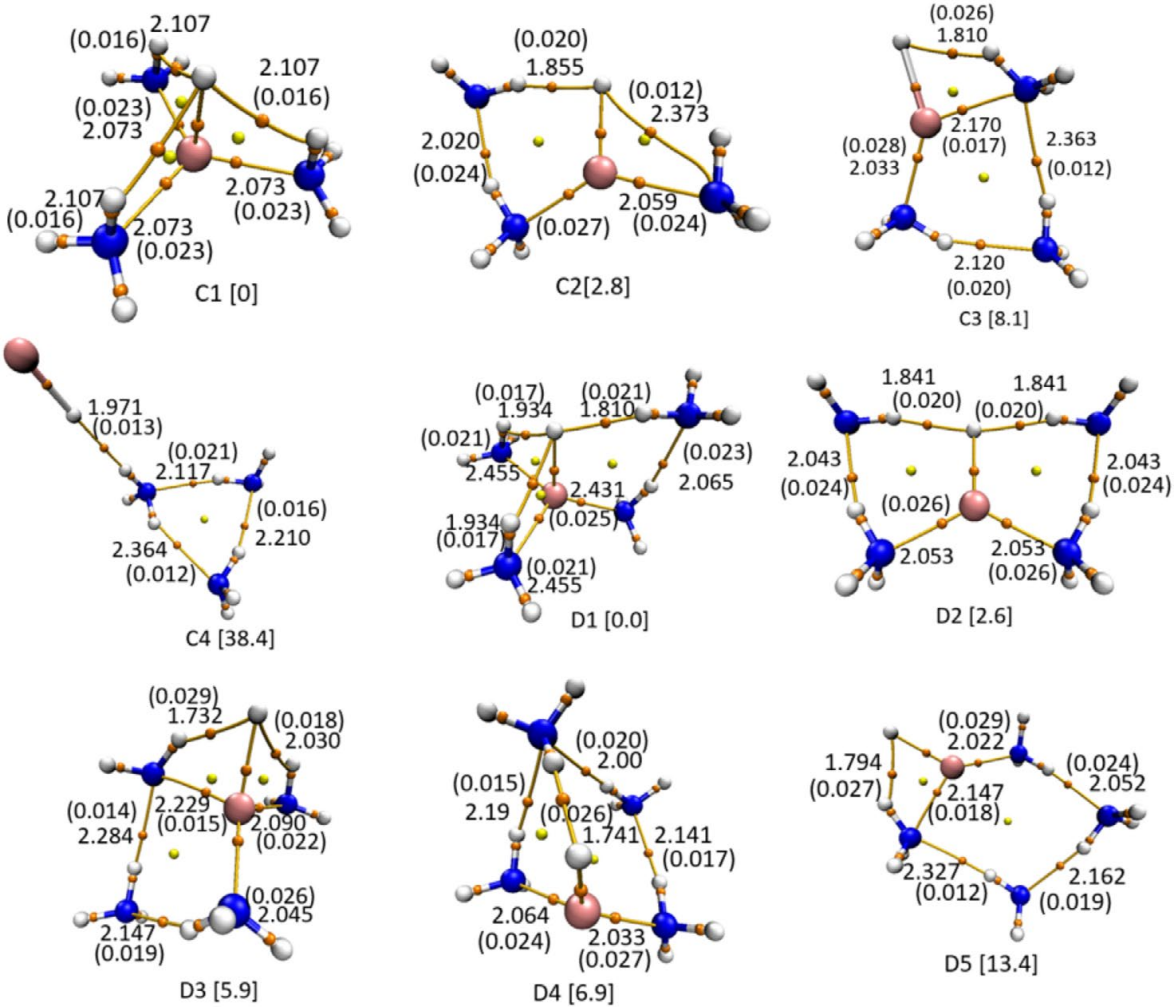
Geometrical conformations of hetero‐tetramer (LiH:3NH_3_) and hetero‐pentamer (LiH:4NH_3_) complexes along with their respective molecular graphs optimized at the RI‐MP2/6‐311++G(2df,2pd) computational level. Values in square brackets are the relative energies (in kcal‐mol^−1^) with respect to their most stable conformer optimized at B3LYP‐D3(BJ)/aug‐cc‐pVDZ. The different legends represent the same meaning as in Figure 2.

This discussion is extended furthermore to understand the complexation behavior in hetero‐tetramer (LiH:3NH_3_) and hetero‐pentamer (LiH:4NH_3_) complexes. The geometrical optimization of the LiH:3NH_3_ complex shows as many as four minima (denoted by C1–C4 in Figure 5), while the LiH:4NH_3_ complex shows five minima (denoted by D1–D5 in Figure 5), of which C1 and D1 represent the global minima where the Li atom adopts a tri‐coordinated configuration (Li···N), while the hydride forms a trifurcated dihydrogen bond (H^−^…H^+^) with ammonia molecules. Additionally, in D1, an extra ammonia molecule forms a hydrogen bond with one of the coordinated ammonia molecules. A close inspection of the C1 conformer shows that each of the three NH_3_ molecules forms a closed structure with LiH, and all these closed structures are fused together via a common LiH molecule. In comparison with B1, C1 shows elongation in R(Li···N) distance (~0.026 Å) leading to a decrease in ρ at the BCP to 0.023 a.u. These details are clearly depicted (Figure 5) in the geometrical structures of their respective conformers, especially the various kinds of cyclic structures in the form of fused rings. From QTAIM plots (shown in Figure S2), it is inferred that both the C3 and B3 configurations resemble each other, with the additional ammonia molecule in C3 interacting via the Li···N and vdW interactions between H^(−)^···N. The conformer C4 has a T‐shaped structure, where the 3‐NH_3_ molecules (forming a cyclic structure) are fused with LiH joining at one of the NH_3_ in the cycle. Thus, C4 adopts an entirely different geometry, characterized only by H···H and N···H interactions. Similar fused structures are also observed for hetero‐pentamer (LiH:4NH_3_) complexes, except for the fact that now the cyclization is much more complicated. Also noticed is that H···H dihydrogen bonds are associated in all the conformers; the conformer D2 is associated with two such dihydrogen bonds, forming a butterfly kind of structure, joined at the LiH molecule. The conformer D3 also has two H···H dihydrogen bonds (joined at LiH molecule) but their bond lengths are markedly different. Whereas D2 has both dihydrogen bonds equal (1.841 Å), the conformer D3 has unequal dihydrogen bonds (1.732 and 2.03 Å). The nature of these interactions, as depicted by the NCI plots (shown in Figure S2) shows characteristic flaps for these two dihydrogen bonds; both green flaps in the case of D2 and one green and another blue flap for D3. Rest all N···H HBs and LBs (N···Li) are as usual. Thus, we understand that the role of HB, DHB, and LB in complexation formed with LiH:3NH_3_ hetero‐tetramer and LiH:4NH_3_ hetero‐pentamer is quite similar to what has been observed in hetero‐dimer (LiH:NH_3_) and hetero‐trimer (LiH:2NH_3_).

### 3.2 Interaction Energy Estimation and Energy Decomposition Analysis

So far, it is observed that among the many geometrical configurations, one particular configuration turns out to be a global minimum, that is, most stable. As why a particular configuration turns out to be the global minimum, is an interesting point to be addressed. The magnitude of interaction energy in different conformers of LiH:nNH_3_ (*n* = 1–4) complexes are greatly influenced by their geometrical structures. In order to understand the origin of non‐additive interaction term induced in cluster systems, decomposition of the interaction energy is carried considering the effects of up to four body interaction terms using methodology described in the computational section. Fragment wise interaction energy calculation of the complexation and the energy decomposition of each of these fragments is undertaken. Initially, the BSSE corrected total interaction energies with RI‐MP2/6‐311++g(2df,2pd)/aug‐cc‐pVTZ, DLPNO‐CCST(T)/aug‐cc‐pVTZ and B3LYP‐D3(BJ)/aug‐cc‐pVDZ/aug‐cc‐pVTZ levels of theories, for the hetero‐dimer (LiH:NH_3_) were estimated. The interaction energy for this hetero dimer as calculated using RI‐MP2, DLPNO‐CCST(T) and DFT level of theory is approximately −19.67/−19.76, −19.93 and −21.40/−21.70 kcal‐mol^−1^ respectively. These values are in close agreement with those reported by Kar et al. (~ −20.0 kcal‐mol^−1^ at MP2/CCSD(T)) [58] and the Andres et al. (−19.5 kcal‐mol^−1^ at B3LYP/aug‐cc‐pVTZ) [59], indicating that the estimate of interaction energy is fairly good by either of the above level of theory. Therefore, in‐depth analysis in terms of the pairwise interaction energy and the energy decomposition, at B3LYP‐D3(BJ)/aug‐cc‐pVDZ level of theory for different conformers of the hetero‐trimer (LiH:2NH_3_) are calculated, as reported in Table 1. As observed from Table 1, the pairwise interaction terms dominating the interaction energy though the non‐additive term, which is in general known as cooperativity, has non‐negligible effect on total energy. Cooperativity $({\Delta E}_{\text{coop}}={\Delta E}_{\mathrm{int}}-$ (E12 + E13 + E23)) as the difference between total interaction energy and the pair wise interaction term is calculated. It is seen that the open structure of B1, B2 shows negative cooperativity (positive values of ${\Delta E}_{\text{coop}}$), while the cyclic structure B3 shows positive cooperativity (negative values of ${\Delta E}_{\text{coop}}$). This cooperative effect is further corroborated with the observed changes in the interatomic distances and QTAIM parameters.

**TABLE 1:** Evaluation of total interaction energy, pair‐wise interaction energy, and other non‐additive interaction energy terms for hetero‐trimer (LiH:2NH_3_). Energies are in kcal‐mol^−1^. For identification of molecules 1, 2, and 3 in B1 and B3, refer to Figure 6b; molecules 1, 2, and 3 in the B2 conformer are identical to those in the B1 conformer. The “Remain” term in the table represents the leftover contributions that are not explicitly accounted for in the many‐body interaction terms.

LiH:2NH_3_	E12	E13	E23	$\Sigma \nabla^{2}E$	Remain	${\Delta E}_{\mathrm{int}}$	${\Delta E}_{\text{coop}}$
B1	2.15	−20.03	−20.04	−37.92	1.14	−36.78	1.14
B2	2.38	−19.91	−20.27	−37.80	1.28	−36.52	1.28
B3	−2.64	−20.79	−7.59	−31.02	−2.58	−33.60	−2.58

The pairwise interaction energy analysis, as mentioned in Table 1, reveals mainly the presence of two dominant interactions; one, the interaction energy associated with each of the H‐Li···N‐H_3_ (AᵢLiH) interactions is approximately 20 kcal‐mol^−1^, and second, the interaction energy associated with each of the NH_3_–NH_3_ (A_i_A_j_) is between 2 and 3 kcal‐mol^−1^. These findings indicate that the stabilization of the LiH:2NH_3_trimer is primarily governed by the attractive (A_i_LiH) interactions. While the (A_i_LiH) interactions are consistently attractive, (A_i_A_j_) interactions are in general repulsive, except for the case of the B3 configuration, where (A_i_A_j_) is attractive due to the prevalent hydrogen bonding. A detailed decomposition of the total interaction energy provides further insight into the underlying mechanisms (see Table 2). In the case of the LiH:NH_3_ dimer, the interaction energy is predominantly governed by electrostatics, followed sequentially by polarization, dispersion, and charge transfer in their increasing contributions. In the LiH:2NH_3_ trimer, while electrostatics and polarization still remain dominant factors, the charge transfer contribution, however, becomes more significant than the dispersion term. The B1 and B2 configurations exhibit negative cooperativity, characterized by their respective non‐additive polarization contributions of ~1.6 kcal‐mol^−1^ and ~1.7 kcal‐mol^−1^, respectively. In contrast to this, the B3 configuration displays positive cooperativity (~ −2.5 kcal‐mol^−1^), with polarization and charge transfer emerging as the primary contributors. This highlights the distinct roles of individual energy components in stabilizing different structural conformations and their influence on cooperativity; at this stage, it is worth mentioning that although the two‐body interaction term is lower in the B3 conformer of the LiH:2NH_3_ cluster compared to B1 and B2, yet the energy component corresponding to the presence of the non‐additive term in B3 provides extra stability. However, its contribution is not sufficient enough to provide the most stable structure, though there is very little difference in their interaction energies (less than 3 kcal‐mol^−1^). Instead, the presence of two Li···N bonds (as explained in previous section) provides extra stability to B1, and hence its global minimum structure.

**TABLE 2:** Decomposition of total interaction energy into five different energy components. Energies are in kcal‐mol^−1^. Description of different energy terms is mentioned in the computational section.

complex	Order	$∆E^{\mathrm{el}}$	$∆E^{\mathrm{xc}}$	$∆E^{\mathrm{pl}}$	$∆E^{\mathrm{ct}}$	$∆E^{\text{disp}}$	$∆E^{\text{BSSE}}$	$∆E^{\mathrm{int}}$
LiH:NH_3_								
A1		−25.86	11.57	−4.80	−2.40	−1.20	1.29	−21.40
LiH:2NH_3_								
B1	2‐body	−60.35	42.82	−12.09	−6.95	−2.90	1.54	−37.93
	Remain	0.00	−0.79	1.59	0.23	0.00	0.120	1.14
	SUM	−60.35	42.03	−10.50	−6.72	−2.90	1.66	−36.78
B2	2‐body	−55.76	36.39	−11.23	−6.00	−2.86	1.67	−37.80
	Remain	0.00	−0.75	1.70	0.33	0.00	0.00	1.28
	SUM	−55.76	35.64	−9.53	−5.67	−2.86	1.67	−36.52
B3	2‐body	−48.81	35.93	−8.31	−8.40	−3.23	1.80	−31.02
	Remain	0.00	0.21	−1.82	−1.06	0.01	0.08	−2.58
	SUM	−48.81	36.14	−10.14	−9.46	−3.21	1.88	−33.60
LiH:3NH_3_								
C1	2‐body	−98.70	83.87	−21.29	−14.32	−5.37	1.88	−53.93
	3‐body	0.00	−2.95	3.75	0.39	−0.05	0.51	1.65
	Remain	0.00	0.10	0.11	−0.05	0.02	0.08	0.26
	SUM	−98.70	81.02	−17.43	−13.98	−5.40	2.47	−52.02
C2	2‐body	−77.13	59.42	−14.59	−12.30	−4.85	2.47	−46.98
	3‐body	0.00	−0.94	−0.42	−1.00	0.02	0.07	−2.42
	Remain	0.00	0.02	0.14	0.11	−0.01	0.03	0.24
	SUM	−77.13	58.5	−14.87	−13.19	−4.84	2.57	−48.96
C3	2‐body	−70.21	55.58	−13.96	−12.92	−5.12	2.73	−43.91
	3‐body	0.00	0.07	0.89	−0.13	0.03	−0.29	0.57
	Remain	0.00	0.02	−0.27	−0.25	−0.01	0.03	−0.48
	SUM	−70.21	55.67	−13.34	−13.30	−5.10	2.47	−43.81
C4	2‐body	−21.33	21.26	−2.92	−8.17	−3.08	2.04	−12.20
	3‐body	0.000	0.13	−0.88	−0.57	0.00	−0.11	−1.43
	Remain	0.000	0.00	0.01	0.00	0.00	0.01	0.02
	SUM	−21.33	21.39	−3.79	−8.74	−3.08	1.94	−13.61
LiH:4NH_3_								
D1	2‐body	−112.13	97.66	−23.49	−19.33	−7.43	2.73	−61.99
	3‐body	0.00	−3.37	1.41	−0.94	0.07	0.35	−2.48
	4‐body	0.00	0.09	0.60	0.38	−0.11	0.09	1.05
	Remain	0.00	−0.01	−0.01	−0.05	0.04	0.02	−0.01
	SUM	−112.13	94.37	−21.49	−19.94	−7.43	3.19	−63.43
D2	2‐body	−92.10	75.25	−16.95	−17.59	−6.81	3.23	−54.97
	3‐body	0.00	−1.38	−2.72	−2.42	0.08	−0.08	−6.52
	4‐body	0.00	−0.04	0.4	0.34	−0.06	0.04	0.68
	Remain	0.00	0.00	0.003	0.00	0.01	0.01	0.02
	SUM	−92.10	73.83	−19.26	−19.67	−6.78	3.20	−60.78
D3	2‐body	−102.37	89.36	−21.96	−19.22	−7.70	3.33	−58.56
	3‐body	0.00	−1.69	3.23	0.23	0.05	−0.06	1.76
	4‐body	0.00	0.11	−0.49	−0.29	−0.07	0.04	−0.70
	Remain	0.00	0.00	0.01	0.03	0.02	0.00	0.06
	SUM	−102.37	87.78	−19.21	−19.25	−7.70	3.31	−57.44
D4	2‐body	−85.24	67.25	−15.89	−16.11	−8.04	3.86	−54.17
	3‐body	0.00	−0.81	−0.50	−0.95	0.08	−0.25	−2.43
	4‐body	0.00	0.07	0.29	−0.13	−0.05	0.04	0.22
	Remain	0.00	−0.01	−0.05	0.02	0.00	0.00	−0.04
	SUM	−85.24	66.50	−16.15	−17.17	−8.01	3.65	−56.45
D5	2‐body	−80.95	67.29	−15.64	−16.04	−6.51	3.21	−48.64
	3‐body	0.00	−0.11	0.09	−0.63	0.04	−0.16	−0.77
	4‐body	0.00	0.01	−0.34	−0.32	−0.02	0.09	−0.58
	Remain	0.00	−0.00	0.00	0.00	0.00	−0.02	−0.02
	SUM	−80.95	67.19	−15.89	−17.00	−6.49	3.12	−50.01

**FIGURE 6:**
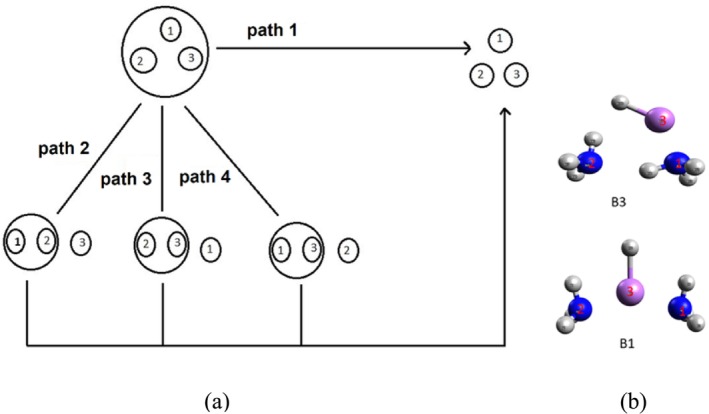
(a) Four possible dissociation paths for LiH:2NH_3_ complex (left); (b) identification of the monomers 1, 2, and 3 pertaining to conformers B1 and B3.

Furthermore, it is worth mentioning that the EDA components can exhibit path dependence and are not uniquely defined, as reported by Diego M. Andrada and Jordi Poater et al. [60, 61, 62]. To assess the extent of path dependency, we performed MB‐EDA based on TSO for the B1 and B3 conformers of the LiH:2NH_3_ complex; needless to mention that the conformers B1 and B2 have nearly identical configurations with nearly similar energies. For each conformer, MB‐EDA calculations were conducted along four different dissociation pathways as shown in Figure 6a. Path 1 shows the dissociation of the hetero‐trimer with three independent monomers. In path 2, the dimer comprising monomer 1 and monomer 2, combined with monomer 3, is dissociated to achieve the independent monomers of the hetero‐trimer. Similar arguments follow for path 3 and path 4 too. Table 3 presents the results of the EDA for different fragmentations of the B1 and B3 complexes for each of the paths as mentioned in Figure 6a. Here, the interaction labeled as 1–2 corresponds to the interaction energy between molecules 1 and 2 in the geometry they adopt in the trimer configuration. Similarly, 12–3 refers to the EDA analysis of molecule 3 interacting with the dimer formed by molecules 1 and 2. Since the preparation (or deformation) energy has been neglected, the absolute value of the interaction energy provides a reasonable approximation of dissociation energy, as shown in Equation (8):

(8) $$\begin{array}{c} -D=∆E^{\text{prep}}+∆E^{\mathrm{int}}\; \end{array}$$

**TABLE 3:** Interaction energy between components of B1 and B3 conformers of LiH:2NH_3_ (kcal‐mol^−1^) optimized at the BLYP‐D3(BJ)/aug‐cc‐pVDZ level of theory. Also given is the MB‐EDA for each of the four paths they follow for dissociation. For identification of 1, 2, and 3 in each conformer, refer to Figure 6b.

Interaction	$∆E^{\mathrm{int}}$	$∆E^{\mathrm{el}}$	$∆E^{\mathrm{xc}}$	$∆E^{\mathrm{pl}}$	$∆E^{\mathrm{ct}}$	$∆E^{\text{disp}}$
Interaction energy between components of conformer B1						
1–2	2.15	2.15	0.38	−0.13	−0.01	−0.23
1–3	−20.05	−31.25	21.23	−5.98	−2.72	−1.34
2–3	−20.06	−31.25	21.21	−5.98	−2.70	−1.34
1–23	−16.74	−28.09	20.29	−4.76	−2.60	−1.57
12–3	−38.94	−59.15	40.36	−12.98	−4.48	−2.68
13–2	−16.75	−28.10	20.27	−4.76	−2.59	−1.57
Paths for the dissociation of B1 conformer using monomers 1, 2, and 3						
Path 1	−36.78	−60.35	42.03	−10.49	−5.05	−2.91
Path 2	−36.79	−57	40.74	−13.11	−4.49	−2.91
Path 3	−36.79	−59.34	41.5	−10.74	−5.30	−2.91
Path 4	−36.79	−59.35	41.5	−10.74	−5.31	−2.91
Interaction energy between components of conformer B3						
1–2	−2.65	−8.21	9.79	−1.13	−2.14	−0.95
1–3	−20.85	−27.25	14.47	−5.18	−1.63	−1.25
2–3	−7.55	−13.35	11.68	−2.00	−2.85	−1.03
1–23	−26.02	−37.28	25.72	−7.77	−4.50	−2.19
12–3	−30.96	−43.25	27.02	−7.88	−4.59	−2.27
13–2	−12.82	−22.92	22.35	−4.16	−6.13	−1.97
Paths for the dissociation of B3 conformer using monomers 1, 2, and 3						
Paths 1	−33.61	−48.81	36.15	−10.14	−7.58	−3.22
Paths 2	−33.61	−51.46	36.81	−9.01	−6.73	−3.22
Paths 3	−33.60	−50.63	37.407	−9.7	−7.35	−3.22
Paths 4	−33.60	−50.17	36.82	−9.34	−7.76	−3.22

Also, given in Table 2 is the value of $∆E^{\mathrm{int}}$ (~ *‐D*) required for dissociating the trimers B1 and B3 into individual molecules via the four different pathways; for instance, in path 2, the interaction energy is obtained by summing the results of 12–3 and 1–2 components, while Path 1, corresponds to the complete dissociation of the trimer into free molecules. In the case of Paths 2, one might expect different contributions from electrostatics, exchange‐correlation, polarization (due to varying dipole orientations), and orbital interactions (i.e., charge transfer) but for the whole paths 123 → 12–3 → 1 + 2 + 3, one would expect no differences in $∆E^{\mathrm{int}},∆E^{\mathrm{el}},∆E^{\mathrm{xc}},∆E^{\mathrm{pl}}$, $∆E^{\mathrm{ct}},∆E^{\text{disp}}$. However, considering the full dissociation process (123 → 12–3 → 1 + 2 + 3), the total interaction energy (ΔE^int^) and dispersion energy (ΔE^disp^) remain consistent across all paths, and thus they refer to as the state functions. Conversely, the exchange‐ correlation (ΔEˣᶜ), polarization (ΔE^pl^), and charge transfer (ΔE^ct^) components are path‐dependent, as confirmed by the results in Table 3 for both the conformers, namely B1 and B3. Nevertheless, the differences among these energy components for different dissociation paths are relatively small (not exceeding 3 kcal‐mol^−1^ and in most of cases less than 1 kcal‐mol^−1^ for charge transfer and polarization). Since the nature of interaction remain consistent across all the path, thus the path dependency of the MB‐EDA method in this particular case study for all practical purposes can be ignored and MB‐EDA has been evaluated using path 1, itself.

To gain a deeper understanding of the interactions within the LiH:nNH_3_ (*n* = 3–4) clusters, we analyzed the effects of cooperativity among the various interactions at play by analyzing the additive and non‐additive energy terms. Total interaction energies were evaluated to capture the collective influence of these cooperative interactions on the system's stability pertaining to various conformers. These energies were decomposed into contributions originating from various types of interaction, including ammonia–ammonia (A_*i*_A_*j*_), ammonia–lithium hydride (A_*i*_LiH), three‐body ammonia–ammonia–ammonia (A_*i*_A_*j*_A_*k*_), ammonia–ammonia–lithium hydride (A_*i*_A_*j*_LiH) and various four‐body systems like ammonia–ammonia–ammonia–ammonia (A_*i*_A_*j*_A_*k*_A_*l*_) and ammonia– ammonia–ammonia–lithium hydride (A_i_A_j_A_k_LiH), as summarized in Table 4 and Table S4a. For the hetero‐tetramer (LiH:3NH_3_), the results indicate that the (AiLiH) interactions are consistently attractive, with their magnitude decreasing from configuration C1 to C4. The two‐body (A_*i*_A_*j*_) interactions are repulsive for C1 and C2 but become attractive for C3 and C4, contributing approximately 68% of the total interaction energy in C4. Significant three‐body (A_*i*_A_*j*_LiH) contributions are also observed, being repulsive for C1 but attractive for C2. The (A_*i*_A_*j*_A_*k*_) interactions are generally small, except for C3 and C4 where they contribute additively towards the total interaction energy. In the case of C1, it is observed that when LiH interacts with an ammonia monomer, there is an electronic charge transfer from the ammonia molecule (primarily from lone pairs on nitrogen) to LiH, resulting in a strong interaction between them. Consequently, it is inferred that in clusters with a small number of ammonia molecules, the system prefers to form as many coordination bonds with lithium as possible, rather than forming hydrogen bonds. This charge transfer further reduces the ability of the Li to act as an electron density acceptor, subsequently weakening the N···Li interactions, resulting in an anti‐cooperative effect. In spite of this, the addition of a larger number of Li···N bonds, as happened in the case of B1, between the monomers provides extra stability.

**TABLE 4:** Many‐body interaction energy analysis and the cooperativity in hetero‐tetramer and pentamer structures. Σ∇^n^ *E* represents the *n*th order interaction. Energies are given in kcal‐mol^−1^.

Complex	$\Sigma \nabla^{2}E$	$\Sigma \nabla^{3}E$	$\Sigma \nabla^{4}E$	Remain	${\Delta E}_{\mathrm{int}}$	${\Delta E}_{\text{coop}}$
Hetero‐tetramer (LiH:3NH_3_)						
C1	−53.93	1.65	—	0.26	−52.02	1.91
C2	−46.98	−2.42		0.24	−48.96	−2.18
C3	−43.91	0.57		−0.48	−43.81	0.09
C4	−12.20	−1.43		0.02	−13.61	−1.41
Hetero‐pentamer (LiH:4NH_3_)						
D1	−61.99	−2.48	1.05	−0.01	−63.43	−1.44
D2	−54.97	−6.52	0.68	0.02	−60.78	−5.82
D3	−58.56	1.76	−0.70	0.06	−57.44	1.12
D4	−54.17	−2.43	0.22	−0.04	−56.45	−2.25
D5	−48.64	−0.77	−0.58	−0.02	−50.01	−1.37

These arguments are further extended to the hetero‐pentamer (LiH:4NH_3_), where (A_*i*_LiH) interactions still remain attractive. In contrast, the (A_*i*_A_*j*_) interactions exhibit maximum repulsion and attraction in D1 and D5 structures, respectively, compared to the tetramer structure. The (A_*i*_A_*j*_LiH) contributions are more significant in the hetero‐pentamer (LiH:4NH_3_) than in the hetero‐tetramer (LiH:3NH_3_), while the (A_*i*_A_*j*_A_*k*_) interactions remain relatively minor. Similarly, the four‐body contributions, (A_*i*_A_*j*_A_*k*_A_*l*_) and (A_*i*_A_*j*_A_*k*_LiH), provide only marginal contributions to the total interaction energy. Krzysztof et al. found that for lithium ion ammonia clusters, the higher order contribution to the total interaction energy is repulsive [63]. However, this study, shows both the attractive as well as repulsive nature. This is possibly due to the interplay between the LB, HB and the DHB. The significant contributions of non‐additive energy terms, particularly (A_*i*_A_*j*_LiH), and (A_*i*_A_*j*_A_*k*_) highlight the cooperative nature of the interactions in the system. This cooperativity is further supported by their geometrical parameters. For the hetero‐pentamer (LiH:4NH_3_), as observed from Table 4, the D2 configuration exhibits the highest cooperativity (−5.82 kcal‐mol^−1^) as the structure is symmetric with respect to central LiH and each ammonia molecule behaving as donor acceptor pair, the energy term is mostly driven by the three‐body contributions ($\Sigma \nabla^{3}E$). Key contributions arise from molecular fragments 1. 2. 5. and 3. 4. 5. (see Table S4a), having equal energy contributions towards positive cooperativity (~ −3.86 kcal‐mol^−1^). Conversely, the D3 configuration shows the negative cooperativity (~ 1.12 kcal‐mol^−1^), primarily due to interactions within the molecular fragments 2. 4. 5. (~ 1.16 kcal‐mol^−1^), where the fourth ammonia acts as a double donor and LiH acts as an acceptor. These results emphasize the role of LB, HB, and DHB in modulating the system's cooperativity and stabilizing the complex. The variation in the different energy component terms, that is, $∆E^{\mathrm{el}}$, $∆E^{\mathrm{xc}}$, $∆E^{\mathrm{pl}}$, $∆E^{\mathrm{ct}}$ and $∆E^{\text{disp}}$ with the number of ammonia molecules and their conformers are tabulated in Table 2. In hetero‐tetramer (LiH:3NH_3_) and hetero‐pentamer (LiH:4NH_3_), the electrostatic and dispersion interactions are pairwise additive, the 2‐body interaction terms for $∆E^{\mathrm{pl}}$, $∆E^{\mathrm{xc}}$ and $∆E^{\mathrm{ct}}$ having significant values. Their 3‐body interaction terms also do have a non‐negligible cooperative influence. The contribution from $∆E^{\mathrm{el}}$ is quite understandable because most of cluster are stabilized by the Li···N bonds in which Li is highly electropositive and N is highly electronegative. The conformers, C4 and D2, do show highest non‐additive term (~ 10% of their total interaction energy), it is observed that main contributions arise from charge transfer and polarization terms, which can also be inferred from their respective geometries and QTAIM parameters, as discussed in previous section. As in C4, where the charge is delocalized through the hydrogen bond, same arguments can be made for the D4 conformer too. Thus, the interaction energy estimation and the EDA of these complexes is able to unfold nature of different kinds of noncovalent bonds and their respective magnitude, further elucidating their interplay in the complexation.

## 4 Summary and Conclusion

A detailed investigation has been conducted to elucidate the nature of lithium hydride–ammonia (LiH–NH_3_) complexation. Computational studies were performed on clusters comprising up to four ammonia (NH_3_) molecules, interacting with a lithium hydride (LiH) molecule. The global and local minimum structures of the LiH:nNH_3_ (*n* = 1–4) clusters were determined using high‐level density functional theory (DFT) and MP2 calculations. These calculations revealed distinct interactions, including Li···N, H···H, and N···H. As the number of ammonia molecules increases, the Li atom adopts a tri‐coordinated (Li···N) configuration (hetero‐tetramer and pentamer), while the hydride forms a trifurcated dihydrogen bond (H^−^···H^+^) with the surrounding ammonia molecules. These interactions were further confirmed through topological analysis using the QTAIM. Employing other descriptors in addition to the BCP, namely, the delocalization index, NCI plots, and EDD mapping augments our understanding of physical interaction in LiH:nNH_3_ complexation. The analysis of hetero‐trimeric, tetrameric, and pentameric systems highlighted a cooperative interplay between hydrogen bonding, DHB, and LB. This interplay was corroborated by examining the geometrical parameters of the clusters. In LiH:2NH_3_ hetero‐trimer, B1 and B2 conformers show A_i_LiH and A_i_A_j_ pairwise attractive and repulsive interaction, but in B3 conformer A_i_A_j_ is found to have pairwise attractive interaction. Many‐body interaction energy calculations revealed that while the clusters are primarily stabilized by two‐body interactions, contributions from other higher body interactions are also significant, for instance, in the D2 conformer of LiH:4NH_3_ and the C4 conformer of LiH:3NH_3_, non‐additive interactions contribute more than ~10%. Among these higher order terms, three‐body interactions are the most prominent ones, highlighting their role in the cooperativity. Furthermore, it is also observed that in hetero‐trimers, open conformers exhibit anti‐cooperative behavior, whereas cyclic conformers, for example, B3 demonstrate cooperative interactions. Among the hetero‐trimers, even though, B3 being a closed structure doesn't form the global minima; instead B1 being the open structure has the global minimum. This is because in B1 two Li···N bonds are present, whereas, only one Li···N bond is present in B3. Here, the Li···N stabilizes the structure of B1 more favorably. Many‐body energy decomposition analyses into five chemically meaningful terms, that is, $∆E^{\mathrm{el}}$, $∆E^{\mathrm{xc}}$, $∆E^{\mathrm{pl}}$, $∆E^{\mathrm{ct}}$, and $∆E^{\text{disp}}$ provided further insights into the origins of cooperative stabilization, revealing that charge transfer and polarization terms do play significant roles. In this study, we find, that although, the cooperativity is observed in less stable compounds, but its effect is significant enough in providing extra stability to it, so that they also become competitive with respect to their most stable counterparts, for example, the difference in the interaction energy between B1, B3; C1, C2; and D1, D2 conformers is less than 3 kcal‐mol^−1^. Thus, by providing additional little amount of extra energy, these relatively less stable conformers can also be arrested (e.g., in exploring their behavior/properties using matrix assisted spectroscopic techniques), which might be of some interest to molecular science. This study highlights the critical role of hydrogen, dihydrogen and Lithium bonds in aggregation of LiH:nNH_3_ clusters and enhances the understanding of inter‐molecular interactions in systems with multiple noncovalent bonds. These findings have potential implications in development of hydrogen production schemes and in materials for hydrogen storage.

## Supporting information


**Data S1:** jcc70114‐sup‐0001‐Supinfo.doc.

## Data Availability

The data that support the findings of this study are available on request from the corresponding author. The data are not publicly available due to privacy or ethical restrictions.
