# Supplementary material for: Computational Study of Complexation in LiH:nNH3 (n = 1–4) Clusters: An Interplay Among Hydrogen, Dihydrogen, and Lithium Bonds
Source: J Comput Chem. 2025 Apr 19;46(11):e70114. doi: 10.1002/jcc.70114 (PMC12008738; doi:10.1002/jcc.70114)
Supplement: Supplementary file 1 — Data S1: jcc70114‐sup‐0001‐Supinfo.doc. [file JCC-46-0-s001.doc]

Supplementary Material

**Computational Study of Complexation in LiH:nNH3 (n=1-4) clusters: An interplay between Hydrogen, Dihydrogen and Lithium Bond**

**Krishna1**, L.K. Saini1, Mukesh Pandey2*

*1Dept. of Physics, Sardar Vallabhbhai National Institute of Technology, Surat - 395007, INDIA*

*2Atomic and Molecular Physics Division, Bhabha Atomic Research Centre, Mumbai - 400085, INDIA*

(*mpandey@barc.gov.in)

**Contents:**

**Table S1**: Bond lengths of most stable structure of LiH:nNH3 (n=1-4) cluster (A1, B1, C1 and D1) optimized at RI-MP2 and B3LYP level of theory with different basis set.

**Table S2:** Quantum theory of Atoms in Molecule Parameters at BCP and the delocalization index of different conformer of LiH:nNH3 (n=1-4) clusters optimized using RI-MP2/ 6-311++g(2df,2pd), Red Colour (Li---H), Green colour (H---H), Blue colour (Li---N), Violet colour (N---H).

**Figure S1:** Different local minima configuration (A: with interatomic distances H···H(-) ~ 1.843 Å (favourable for DHB); B: with N···H(-) distance ~1.863 Å (favourable for HB) and; C: with N···H(-) distance ~ 1.686 Å) of LiH:NH3 as obtained from ABCluster (left) and final configuration after optimization optimized at RI-MP2/6-311++g(2df,2pd) (right).

**Figure S2:** AIM+NCI picture of LiH:nNH3 (n=1-4) conformers, coral-pink colour refers to lithium atom, blue refers to Nitrogen and silver-white colour refers to the hydrogen atom. The different conformers– 1) A1 corresponds to LiH:NH3, 2) B1, B2, B3 corresponds to LiH:2NH3 3) C1, C2, C3 and C4 corresponds to LiH:3NH3, 4) D1, D2, D3, D4 and D5 corresponds to LiH:4NH3

**Table S3**: Table S3: AIM charge of different conformers of LiH:nNH3 (n=1-4) optimized at RI-MP2/ 6-311++g(2df,2pd)

**Table S4:** Many Body Energy contribution to total interaction energy up-to four body (Kcal-mol-1) for different conformer of LiH:nNH3 (n=1-4) clusters

**Table S4a**: Fragment wise interaction energy calculation of different conformer of LiH:nNH3 (n=1-4) clusters optimized at B3LYP/aug-cc-pVDZ (Kcal-mol-1). Figures of different conformers identifying the associated fragments are also given alongside with identification number of the fragments. Also shown is the decomposition of interaction energy into contribution from various interaction type (A*i*A*j*), ammonia–lithium hydride (A*i*LiH), three-body ammonia–ammonia–ammonia (A*i*A*j*A*k*), ammonia–ammonia–lithium hydride (A*i*A*j*LiH), four-body ammonia–ammonia–ammonia–ammonia (A*i*A*j*A*k*A*l*), and ammonia–ammonia–ammonia–lithium hydride (A*i*A*j*A*k*LiH).

**Coordinates:** The XYZ coordinates of LiH:nNH3 (n=1-4)

**Table S1: Bond lengths of most stable structure of LiH:nNH3 (n=1-4) cluster (A1, B1, C1 and D1) optimized at RI-MP2 and B3LYP level of theory with different basis set.**

| Atoms | Bond length /Å | Atoms | Bond length /Å | Atoms | Bond length /Å | Atoms | Bond length /Å |
| --- | --- | --- | --- | --- | --- | --- | --- |
| **RI-MP2/** **6-311++g(2df,2pd)** |  | **RI-MP2/aug-cc-pVTZ** |  | **B3LYP/aug-cc-pVDZ** |  | **B3LYP/** **6-311++g(2df,2pd)** |  |
| LiH:NH3 |  |  |  |  |  |  |  |
| N0–H1  N0–H2  N0–H3  N0–Li4  Li4–H5 | 1.0121  1.0121  1.0121  2.0479  1.6203 | N0–H1  N0–H2  N0–H3  N0–Li4  Li4–H5 | 1.0116  1.0116  1.0116  2.0328  1.6181 | N0–H1  N0–H2  N0–H3  N0–Li4  Li4–H5 | 1.0206  1.0205  1.0206  2.0495  1.6229 | N0–H1  N0–H2  N0–H3  N0–Li4  Li4–H5 | 1.0155  1.0155  1.0155  2.0383  1.6201 |
| LiH:2NH3 |  |  |  |  |  |  |  |
| N0–H1  N0–H2  N0–H3  N0–Li8  N4–H5  N4–H6  N4–H7  N4–Li8  Li8–H9 | 1.0105  1.0106  1.0157  2.0466  1.0106  1.0105  1.0156  2.0466  1.6790 | N0–H1  N0–H2  N0–H3  N0–Li8  N4–H5  N4–H6  N4–H7  N4–Li8  Li8–H9 | 1.0100  1.0100  1.0167  2.0341  1.0100  1.0100  1.0167  2.0341  1.6873 | N0–H1  N0–H2  N0–H3  N0–Li8  N4–H5  N4–H6  N4–H7  N4–Li8  Li8–H9 | 1.0188  1.0188  1.0253  2.0562  1.0188  1.0188  1.0252  2.0561  1.6922 | N0–H1  N0–H2  N0–H3  N0–Li8  N4–H5  N4–H6  N4–H7  N4–Li8  Li8–H9 | 1.0139  1.0140  1.0196  2.0511  1.0140  1.0140  1.0196  2.0511  1.6868 |
| LiH:3NH3 |  |  |  |  |  |  |  |
| N0–H1  N0–H2  N0–H3  N0–Li12  N4–H5  N4–H6  N4–H7  N4–Li12  N8–H9  N8–H10  N8–H11  N8–Li12 | 1.0103  1.0211  1.0103  2.0730  1.0103  1.0103  1.0211  2.0730  1.0103  1.0103  1.0211  2.0730 | N0–H1  N0–H2  N0–H3  N0–Li12  N4–H5  N4–H6  N4–H7  N4–Li12  N8–H9  N8–H10  N8–H11  N8–Li12 | 1.0097  1.0219  1.0097  2.0599  1.0097  1.0097  1.0219  2.0599  1.0097  1.0097  1.0219  2.0599 | N0–H1  N0–H2  N0–H3  N0–Li12  N4–H5  N4–H6  N4–H7  N4–Li12  N8–H9  N8–H10  N8–H11  N8–Li12 | 1.0183  1.0307  1.0183  2.0857  1.0183  1.0183  1.0307  2.0856  1.0183  1.0183  1.0307  2.0855 | N0–H1  N0–H2  N0–H3  N0–Li12  N4–H5  N4–H6  N4–H7  N4–Li12  N8–H9  N8–H10  N8–H11  N8–Li12 | 1.0135  1.0252  1.0135  2.0903  1.0135  1.0135  1.0252  2.0904  1.0135  1.0135  1.0252  2.0904 |
| LiH:4NH3 |  |  |  |  |  |  |  |
| N0–H1  N0–H2  N0–H3  N0–Li16  N4–H5  N4–H6  N4–H7  N8–H9  N8–H10  N8–H11  N8–Li16  N12–H13  N12–H14  N12–H15  N12–Li16 | 1.0109  1.0247  1.0109  2.0564  1.0114  1.0288  1.0114  1.0103  1.0220  1.0103  2.0972  1.0103  1.0103  1.0220  2.0972 | N0–H1  N0–H2  N0–H3  N0–Li16  N4–H5  N4–H6  N4–H7  N8–H9  N8–H10  N8–H11  N8–Li16  N12–H13  N12–H14  N12–H15  N12–Li16 | 1.0107  1.0242  1.0107  2.0467  1.0114  1.0279  1.0114  1.0096  1.0223  1.0098  2.0837  1.0096  1.0098  1.0223  2.0837 | N0–H1  N0–H2  N0–H3  N0–Li16  N4–H5  N4–H6  N4–H7  N8–H9  N8–H10  N8–H11  N8–Li16  N12–H13  N12–H14  N12–H15  N12–Li16 | 1.0187  1.0336  1.0187  2.0724  1.0192  1.0380  1.0193  1.0183  1.0319  1.0182  2.1114  1.0183  1.0182  1.0319  2.1114 | N0–H1  N0–H2  N0–H3  N0–Li16  N4–H5  N4–H6  N4–H7  N8–H9  N8–H10  N8–H11  N8–Li16  N12–H13  N12–H14  N12–H15  N12–Li16 | 1.0140  1.0281  1.0139  2.0721  1.0143  1.0322  1.0143  1.0135  1.0264  1.0135  2.1187  1.0135  1.0134  1.0263  2.1181 |

**Table S2: Quantum theory of Atoms in Molecule Parameters at BCP and the delocalization index of different conformer of LiH:nNH3 (n=1-4) clusters optimized using RI-MP2/ 6-311++g(2df,2pd), Red Colour (Li---H), Green colour (H---H), Blue colour (Li---N), Violet colour (N---H).**

| connecting atom | electron density | kinetic energy G(r) | Potential energy  V(r) | Total energy  E(r) | Laplacian of electron density | Delocalization index (DI) |
| --- | --- | --- | --- | --- | --- | --- |
| LiH:NH­3 |  |  |  |  |  |  |
| 6(H ) -- 5(Li) | 3.76E-02 | 3.79E-02 | -3.86E-02 | -0.750E-03 | 1.49E-01 | 0.932 |
| 5(Li) -- 1(N) | 2.70E-02 | 3.47E-02 | -2.86E-02 | 6.12E-03 | 1.63E-01 | 0.447 |
| LiH:2NH3 |  |  |  |  |  |  |
| B1 |  |  |  |  |  |  |
| 5(N ) -- 9(Li) | 2.54E-02 | 3.39E-02 | -2.74E-02 | 6.49E-03 | 1.62E-01 | 0.444 |
| 10(H) -- 9(Li) | 3.33E-02 | 3.20E-02 | -3.29E-02 | -0.841E-03 | 1.25E-01 | 0.806 |
| 9(Li) -- 1(N) | 2.54E-02 | 3.39E-02 | -2.74E-02 | 6.49E-03 | 1.62E-01 | 0.444 |
| B2 |  |  |  |  |  |  |
| 5(N ) -- 9(Li) | 2.57E-02 | 3.40E-02 | -2.76E-02 | 6.41E-03 | 1.62E-01 | 0.451 |
| 9(Li) -- 10(H) | 3.36E-02 | 3.23E-02 | -3.31E-02 | -8.46E-04 | 1.26E-01 | 0.813 |
| 9(Li) -- 1(N) | 2.48E-02 | 3.33E-02 | -2.68E-02 | 6.49E-03 | 1.59E-01 | 0.443 |
| 10(H) -- 1(N) | 1.22E-02 | 6.91E-03 | -7.39E-03 | -4.80E-04 | 2.57E-02 | 0.026 |
| B3 |  |  |  |  |  |  |
| 10(H) -- 6(H) | 1.82E-02 | 8.78E-03 | -9.52E-03 | -7.41E-04 | 3.21E-02 | 0.110 |
| 10(H) -- 9(Li) | 3.60E-02 | 3.57E-02 | -3.65E-02 | -7.63E-04 | 1.40E-01 | 0.807 |
| 9(Li) -- 1(N) | 2.96E-02 | 3.92E-02 | -3.26E-02 | 6.57E-03 | 1.83E-01 | 0.466 |
| 5(N) -- 4(H) | 2.45E-02 | 1.69E-02 | -1.70E-02 | -1.71E-04 | 6.68E-02 | 0.090 |
| LiH:3NH­3 |  |  |  |  |  |  |
| C1 |  |  |  |  |  |  |
| 3(H) -- 14(H) | 1.61E-02 | 7.99E-03 | -8.26E-03 | -0.270E-03 | 3.09E-02 | 0.081 |
| 1(N) -- 13(Li) | 2.33E-02 | 3.11E-02 | -2.49E-02 | 6.22E-03 | 1.49E-01 | 0.418 |
| 14(H) -- 13(Li) | 2.48E-02 | 2.37E-02 | -2.37E-02 | 0.0161E-03 | 9.48E-02 | 0.594 |
| 14(H) -- 8(H) | 1.61E-02 | 7.99E-03 | -8.26E-03 | -0.270E-03 | 3.09E-02 | 0.081 |
| 14(H) -- 12(H) | 1.61E-02 | 7.99E-03 | -8.26E-03 | -0.270E-03 | 3.09E-02 | 0.081 |
| 13(Li) -- 5(N) | 2.33E-02 | 3.11E-02 | -2.49E-02 | 6.22E-03 | 1.49E-01 | 0.418 |
| 13(Li) -- 9(N) | 2.33E-02 | 3.11E-02 | -2.49E-02 | 6.22E-03 | 1.49E-01 | 0.418 |
| C2 |  |  |  |  |  |  |
| 10(H) -- 5(N) | 2.49E-02 | 1.73E-02 | -1.77E-02 | -3.45E-04 | 6.79E-02 | 0.092 |
| 9(N) -- 13(Li) | 2.72E-02 | 3.55E-02 | -2.93E-02 | 6.27E-03 | 1.67E-01 | 0.453 |
| 13(Li) -- 1(N) | 2.44E-02 | 3.26E-02 | -2.62E-02 | 6.38E-03 | 1.56E-01 | 0.433 |
| 13(Li) -- 14(H) | 3.12E-02 | 2.99E-02 | -3.04E-02 | -4.57E-04 | 1.18E-01 | 0.710 |
| 1(N) -- 14(H) | 1.22E-02 | 6.71E-03 | -7.13E-03 | -4.14E-04 | 2.52E-02 | 0.023 |
| 8(H) -- 14(H) | 2.04E-02 | 9.86E-03 | -1.11E-02 | -1.25E-03 | 3.44E-02 | 0.130 |
| C3 |  |  |  |  |  |  |
| 9(N) -- 3(H) | 2.07E-02 | 1.48E-02 | -1.40E-02 | 8.77E-04 | 6.28E-02 | 0.077 |
| 11(H) -- 5(N) | 1.24E-02 | 8.49E-03 | -7.26E-03 | 1.23E-03 | 3.89E-02 | 0.046 |
| 1(N) -- 13(Li) | 2.82E-02 | 3.65E-02 | -3.03E-02 | 6.21E-03 | 1.71E-01 | 0.451 |
| 5(N) -- 13(Li) | 1.78E-02 | 2.41E-02 | -1.88E-02 | 5.29E-03 | 1.18E-01 | 0.350 |
| 7(H) -- 14(H) | 2.62E-02 | 1.22E-02 | -1.47E-02 | -2.52E-03 | 3.87E-02 | 0.136 |
| 13(Li) -- 14(H) | 3.29E-02 | 3.19E-02 | -3.25E-02 | -5.88E-04 | 1.25E-01 | 0.749 |
| C4 |  |  |  |  |  |  |
| 14(H) -- 3(H) | 1.31E-02 | 7.23E-03 | -6.86E-03 | 3.65E-04 | 3.04E-02 | 0.102 |
| 12(H) -- 1(N) | 2.10E-02 | 1.47E-02 | -1.41E-02 | 5.99E-04 | 6.10E-02 | 0.082 |
| 9(N) -- 6(H) | 1.68E-02 | 1.19E-02 | -1.07E-02 | 1.25E-03 | 5.28E-02 | 0.067 |
| 2(H) -- 5(N) | 1.22E-02 | 8.66E-03 | -7.18E-03 | 1.48E-03 | 4.06E-02 | 0.051 |
| 13(Li) -- 14(H) | 4.03E-02 | 4.25E-02 | -4.31E-02 | -5.88E-04 | 1.68E-01 | 0.843 |
|  |  |  |  |  |  |  |
| LiH:4NH­3 |  |  |  |  |  |  |
| D1 |  |  |  |  |  |  |
| 13(N ) -- 17(Li) | 2.17E-02 | 2.89E-02 | -2.29E-02 | 5.95E-03 | 1.39E-01 | 0.401 |
| 16(H ) -- 18(H ) | 1.76E-02 | 8.84E-03 | -9.14E-03 | -0.302E-03 | 3.42E-02 | 0.087 |
| 1(N ) -- 17(Li) | 2.58E-02 | 3.34E-02 | -2.73E-02 | 6.13E-03 | 1.58E-01 | 0.435 |
| 3(H) -- 5(N) | 2.37E-02 | 1.67E-02 | -1.67E-02 | -0.006E-03 | 6.69E-02 | 0.088 |
| 17(Li) -- 18(H) | 2.35E-02 | 2.23E-02 | -2.20E-02 | 0.296E-03 | 9.03E-02 | 0.552 |
| 7(H) -- 18(H) | 2.11E-02 | 1.02E-02 | -1.16E-02 | -1.39E-03 | 3.52E-02 | 0.126 |
| 17(Li) -- 9(N) | 2.17E-02 | 2.89E-02 | -2.29E-02 | 5.95E-03 | 1.39E-01 | 0.401 |
| 18(H) -- 11(H) | 1.76E-02 | 8.84E-03 | -9.14E-03 | -0.302E-03 | 3.42E-02 | 0.087 |
| D2 |  |  |  |  |  |  |
| 15(H) -- 9(N) | 2.45E-02 | 1.72E-02 | -1.74E-02 | -2.41E-04 | 6.79E-02 | 0.091 |
| 13(N) -- 17(Li) | 2.61E-02 | 3.39E-02 | -2.78E-02 | 6.11E-03 | 1.60E-01 | 0.442 |
| 12(H) -- 18(H) | 2.04E-02 | 1.00E-02 | -1.12E-02 | -1.16E-03 | 3.55E-02 | 0.123 |
| 17(Li) -- 18(H) | 2.97E-02 | 2.84E-02 | -2.85E-02 | -7.87E-05 | 1.13E-01 | 0.647 |
| 17(Li) -- 1(N) | 2.61E-02 | 3.39E-02 | -2.78E-02 | 6.11E-03 | 1.60E-01 | 0.442 |
| 18(H) -- 8(H) | 2.04E-02 | 1.00E-02 | -1.12E-02 | -1.16E-03 | 3.55E-02 | 0.123 |
| 2(H) -- 5(N) | 2.45E-02 | 1.72E-02 | -1.74E-02 | -2.39E-04 | 6.79E-02 | 0.091 |
| D3 |  |  |  |  |  |  |
| 1(N ) -- 8(H ) | 1.94E-02 | 1.40E-02 | -1.29E-02 | 1.14E-03 | 6.07E-02 | 0.072 |
| 13(N ) -- 17(Li) | 2.21E-02 | 2.97E-02 | -2.36E-02 | 6.07E-03 | 1.43E-01 | 0.406 |
| 5(N) -- 17(Li) | 2.68E-02 | 3.49E-02 | -2.86E-02 | 6.25E-03 | 1.64E-01 | 0.440 |
| 15(H) -- 18(H) | 1.80E-02 | 9.07E-03 | -9.53E-03 | -4.61E-04 | 3.44E-02 | 0.088 |
| 3(H) -- 9(N) | 1.47E-02 | 1.02E-02 | -8.97E-03 | 1.20E-03 | 4.55E-02 | 0.053 |
| 17(Li) -- 9(N) | 1.53E-02 | 2.05E-02 | -1.60E-02 | 4.56E-03 | 1.00E-01 | 0.313 |
| 17(Li) -- 18(H) | 2.70E-02 | 2.58E-02 | -2.58E-02 | -7.46E-05 | 1.03E-01 | 0.614 |
| 18(H) -- 12(H) | 2.93E-02 | 1.33E-02 | -1.69E-02 | -3.58E-03 | 3.88E-02 | 0.147 |
| D4 |  |  |  |  |  |  |
| 16(H) -- 5(N) | 1.58E-02 | 1.17E-02 | -1.02E-02 | 1.45E-03 | 5.24E-02 | 0.055 |
| 4(H) -- 5(N) | 1.72E-02 | 1.20E-02 | -1.10E-02 | 1.01E-03 | 5.19E-02 | 0.065 |
| 13(N) -- 17(Li) | 2.47E-02 | 3.25E-02 | -2.63E-02 | 6.22E-03 | 1.55E-01 | 0.420 |
| 1(N) -- 10(H) | 2.02E-02 | 1.39E-02 | -1.32E-02 | 7.23E-04 | 5.87E-02 | 0.075 |
| 17(Li) -- 9(N) | 2.70E-02 | 3.57E-02 | -2.93E-02 | 6.38E-03 | 1.68E-01 | 0.443 |
| 6(H) -- 18(H) | 2.60E-02 | 1.19E-02 | -1.49E-02 | -2.98E-03 | 3.58E-02 | 0.149 |
| 17(Li) -- 18(H) | 3.14E-02 | 3.01E-02 | -3.04E-02 | -3.55E-04 | 1.19E-01 | 0.718 |
| D5 |  |  |  |  |  |  |
| 18(H) -- 17(Li) | 3.22E-02 | 3.11E-02 | -3.17E-02 | -5.94E-04 | 1.22E-01 | 0.738 |
| 1(N) -- 17(Li) | 2.90E-02 | 3.78E-02 | -3.14E-02 | 6.34E-03 | 1.76E-01 | 0.464 |
| 18(H) -- 14(H) | 2.70E-02 | 1.25E-02 | -1.52E-02 | -2.72E-03 | 3.91E-02 | 0.140 |
| 17(Li) -- 13(N) | 1.89E-02 | 2.57E-02 | -2.01E-02 | 5.56E-03 | 1.25E-01 | 0.367 |
| 4(H) -- 5(N) | 2.42E-02 | 1.67E-02 | -1.71E-02 | -3.59E-04 | 6.54E-02 | 0.091 |
| 13(N) -- 10(H) | 1.29E-02 | 9.07E-03 | -7.79E-03 | 1.28E-03 | 4.14E-02 | 0.049 |
| 8(H) -- 9(N) | 1.91E-02 | 1.33E-02 | -1.24E-02 | 8.75E-04 | 5.68E-02 | 0.076 |

**Figure S1: Different local minima configuration LiH:NH3­ as obtained from ABCluster (left) and after optimization at RI-MP2/6-311++g(2df,2pd) (right). Below showing three different initial configurations- A: having H···H(-) distance ~ 1.843 Å (favourable for DHB); B: having N···H(-) distance ~1.863 Å (favourable for HB) and; C: having N···H(-) distance ~ 1.686 Å with H(-) within the ‘3H’ umbrella of NH3. After optimization at RI-MP2/6-311++g(2df,2pd) all three converging into one equivalent configuration.**

| **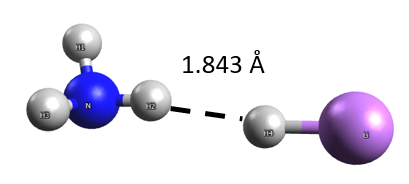**  **A** | **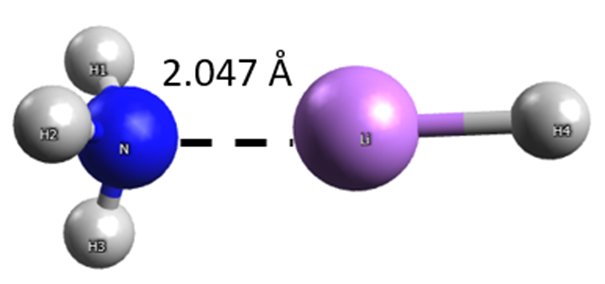** |
| --- | --- |
| **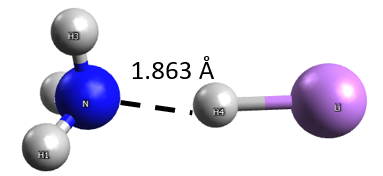**  **B** | **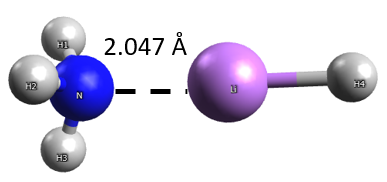** |
| **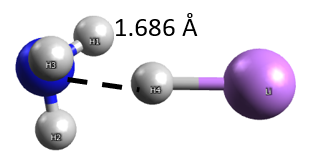**  **C** | **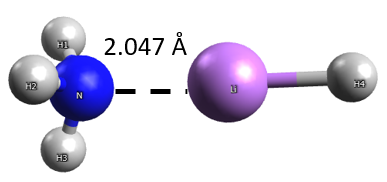** |

**Figure S2: AIM+NCI picture of LiH:nNH3 (n=1-4) conformers, coral-pink colour refers to lithium atom, blue refers to Nitrogen and silver-white colour refers to the hydrogen atom. The different conformers– 1) A1 corresponds to LiH:NH3, 2) B1, B2, B3 corresponds to LiH:2NH3 3) C1, C2, C3 and C4 corresponds to LiH:3NH3, 4) D1, D2, D3, D4 and D5 corresponds to LiH:4NH3.**


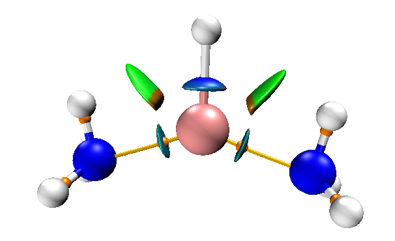

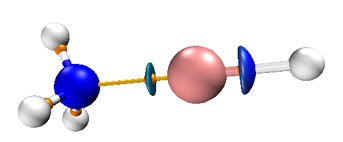

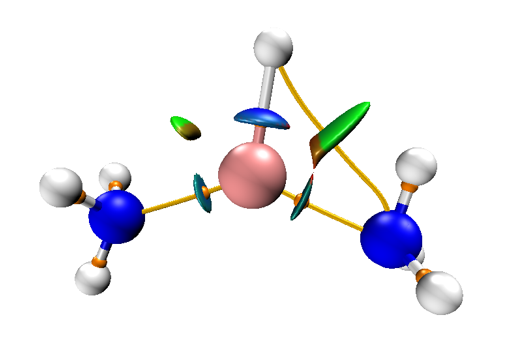

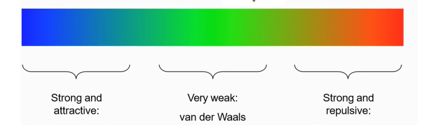


| **A1** | **B1** | **B2** |
| --- | --- | --- |

**
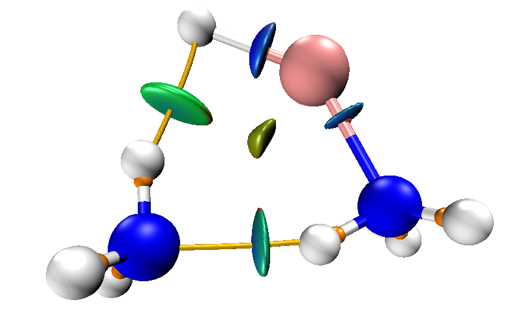

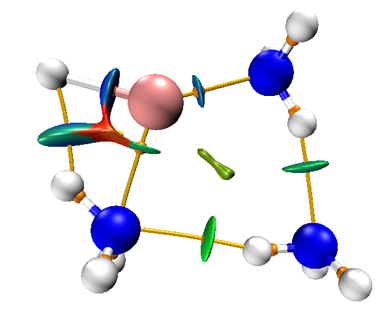
**

**
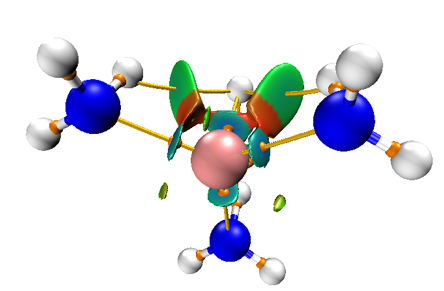

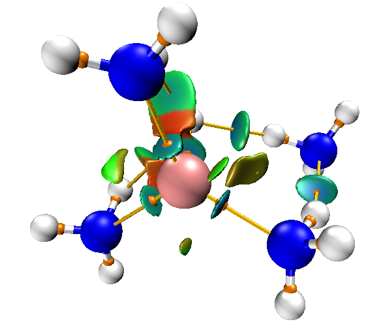

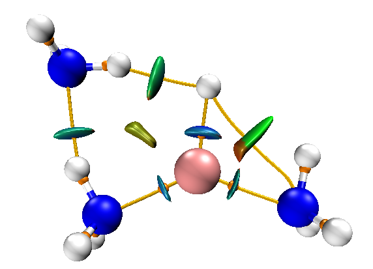

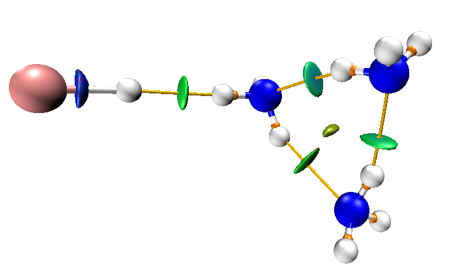
**

| **B3** | **C1** | **C2** |  |
| --- | --- | --- | --- |

| **C3** | **C4** | **D1** |
| --- | --- | --- |

**
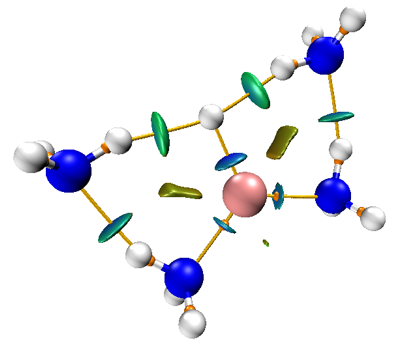

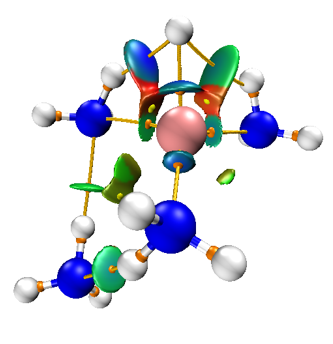

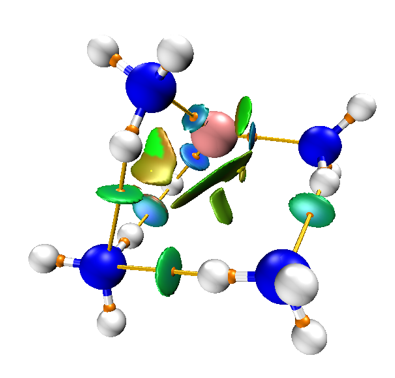
**

| **D2** | **D3** | **D4** |
| --- | --- | --- |

**
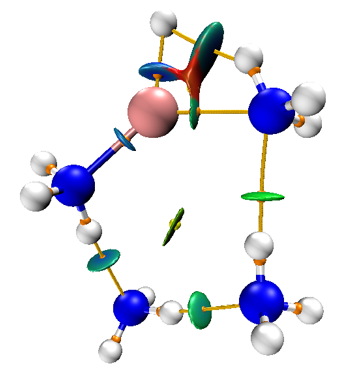
**

| **D5** |  |  |
| --- | --- | --- |

**Table S3: AIM charge of different conformers of LiH:nNH3 (n=1-4) optimized at RI-MP2/ 6-311++g(2df,2pd)**

NH3

| N | -1.013 |
| --- | --- |
| H | 0.381 |
| H | 0.380 |
| H | 0.380 |

LiH

| Li | 0.915 |
| --- | --- |
| H | -0.883 |

| LiH:NH3 |  | LiH:2NH3 |  |  |  | LiH:3NH3 |  |  |  |  |
| --- | --- | --- | --- | --- | --- | --- | --- | --- | --- | --- |
| A1 |  | B1 |  | B2 | B3 | C1 |  | C2 | C3 | C4 |
| N1 | -1.093 | N1 | -1.108 | -1.114 | -1.162 | N1 | -1.118 | -1.111 | -1.136 | -1.089 |
| H2 | 0.414 | H2 | 0.398 | 0.396 | 0.397 | H2 | 0.389 | 0.396 | 0.397 | 0.419 |
| H3 | 0.414 | H3 | 0.398 | 0.438 | 0.396 | H3 | 0.443 | 0.437 | 0.466 | 0.427 |
| H4 | 0.414 | H4 | 0.434 | 0.396 | 0.487 | H4 | 0.389 | 0.396 | 0.397 | 0.361 |
| Li5 | 0.894 | N5 | -1.109 | -1.089 | -1.091 | N5 | -1.117 | -1.093 | -1.144 | -1.058 |
| H6 | -0.883 | H6 | 0.398 | 0.398 | 0.448 | H6 | 0.389 | 0.371 | 0.384 | 0.446 |
|  |  | H7 | 0.399 | 0.411 | 0.377 | H7 | 0.389 | 0.371 | 0.458 | 0.367 |
|  |  | H8 | 0.433 | 0.413 | 0.377 | H8 | 0.443 | 0.456 | 0.384 | 0.368 |
|  |  | Li9 | 0.892 | 0.891 | 0.900 | N9 | -1.120 | -1.147 | -1.054 | -1.075 |
|  |  | H10 | -0.844 | -0.848 | -0.834 | H10 | 0.389 | 0.487 | 0.386 | 0.367 |
|  |  |  |  |  |  | H11 | 0.389 | 0.388 | 0.424 | 0.367 |
|  |  |  |  |  |  | H12 | 0.445 | 0.387 | 0.386 | 0.464 |
|  |  |  |  |  |  | Li13 | 0.896 | 0.893 | 0.895 | 0.918 |
|  |  |  |  |  |  | H14 | -0.779 | -0.804 | -0.817 | -0.855 |
|  |  |  |  |  |  |  |  |  |  |  |
| LiH:4NH3 |  |  |  |  |  |  |  |  |  |  |
| D1 |  | D2 | D3 | D4 | D5 |  |  |  |  |  |
| N1 | -1.130 | -1.139 | -1.059 | -1.063 | -1.148 |  |  |  |  |  |
| H2 | 0.383 | 0.485 | 0.381 | 0.376 | 0.397 |  |  |  |  |  |
| H3 | 0.480 | 0.384 | 0.433 | 0.379 | 0.388 |  |  |  |  |  |
| H4 | 0.383 | 0.384 | 0.381 | 0.445 | 0.480 |  |  |  |  |  |
| N5 | -1.091 | -1.091 | -1.121 | -1.109 | -1.072 |  |  |  |  |  |
| H6 | 0.368 | 0.371 | 0.390 | 0.467 | 0.380 |  |  |  |  |  |
| H7 | 0.456 | 0.371 | 0.396 | 0.368 | 0.378 |  |  |  |  |  |
| H8 | 0.368 | 0.456 | 0.459 | 0.368 | 0.453 |  |  |  |  |  |
| N9 | -1.117 | -1.092 | -1.132 | -1.131 | -1.048 |  |  |  |  |  |
| H10 | 0.385 | 0.371 | 0.375 | 0.463 | 0.426 |  |  |  |  |  |
| H11 | 0.445 | 0.372 | 0.377 | 0.386 | 0.382 |  |  |  |  |  |
| H12 | 0.386 | 0.455 | 0.457 | 0.399 | 0.382 |  |  |  |  |  |
| N13 | -1.115 | -1.139 | -1.120 | -1.106 | -1.148 |  |  |  |  |  |
| H14 | 0.385 | 0.384 | 0.386 | 0.395 | 0.460 |  |  |  |  |  |
| H15 | 0.386 | 0.485 | 0.446 | 0.388 | 0.383 |  |  |  |  |  |
| H16 | 0.444 | 0.385 | 0.388 | 0.447 | 0.384 |  |  |  |  |  |
| Li17 | 0.895 | 0.893 | 0.895 | 0.892 | 0.895 |  |  |  |  |  |
| H18 | -0.751 | -0.775 | -0.771 | -0.804 | -0.814 |  |  |  |  |  |

**Table S4: Many Body Energy contribution to total interaction energy up-to four body (kcal-mol-1)** **for different conformer of LiH:nNH3 (n=1-4) clusters.**

| complex | 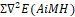 | 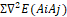 | 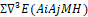 | 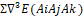 | 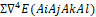 | 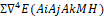 | 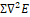 | 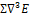 | 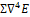 |
| --- | --- | --- | --- | --- | --- | --- | --- | --- | --- |
| | LiH:NH3 | | --- | | -21.40 |  |  |  |  |  | -37.930 |  |  |
| LiH:2NH3 |  |  |  |  |  |  |  |  |  |
| B1 | -40.077 | 2.153 |  |  |  |  | -37.930 |  |  |
| B2 | -40.186 | 2.384 |  |  |  |  |  |  |  |
| B3 | -28.38 | -2.649 |  |  |  |  |  |  |  |
| LiH:3NH3 |  |  |  |  |  |  |  |  |  |
| C1 | -60.384 | 6.44 | 2.132 | -0.487 |  |  | -53.944 | 1.645 |  |
| C2 | -47.023 | 0.036 | -2.712 | 0.288 |  |  | -46.989 | -2.424 |  |
| C3 | -40.714 | -3.199 | 0.160 | -3.981 |  |  | -43.913 | 0.569 |  |
| C4 | -3.031 | -9.189 | -0.111 | -1.225 |  |  | -12.222 | -1.336 |  |
| LiH:4NH3 |  |  |  |  |  |  |  |  |  |
| D1 | -66.65 | 4.652 | -2.513 | 0.043 | -0.037 | 1.109 | -61.998 | -2.470 | 1.072 |
| D2 | -52.903 | -2.088 | -7.274 | 0.75 | 0.62 | 0.072 | -54.992 | -6.522 | 0.693 |
| D3 | -58.901 | 0.332 | 2.684 | -0.92 | -0.758 | 0.057 | -58.569 | 1.764 | -0.701 |
| D4 | -50.425 | -3.745 | -2.412 | -0.067 | 0.148 | 0.07 | -54.17 | -2.479 | 0.218 |
| D5 | -41.756 | -6.878 | 0.479 | -1.248 | -0.587 | 0.002 | -48.636 | -0.767 | -0.587 |

**Table S4a: Fragment wise interaction energy calculation of different conformer of LiH:nNH3 (n=1-4) clusters optimized at B3LYP/aug-cc-pVDZ (kcal-mol-1). Figures of different conformers identifying the associated fragments are given alongside with identification number of these fragments. Also shown is the decomposition of interaction energy into contribution from various interaction type (AiAj), ammonia–lithium hydride (AiLiH), three-body ammonia–ammonia–ammonia (AiAjAk), ammonia–ammonia–lithium hydride (AiAjLiH), four-body ammonia–ammonia–ammonia–ammonia (AiAjAkAl), and ammonia–ammonia–ammonia–lithium hydride (AiAjAkLiH).**

| **Interaction Energy** | **fragments** |
| --- | --- |
| LiH:2NH3 | |
| B1 |  |
| 2.153  -20.037  -20.047 | 1.2.  1.3.  2.3. |
| -37.930 |  |
| B2 |  |
| 2.384  -19.912  -20.274 | 1.2.  1.3.  2.3. |
| -37.802 |  |
| B3 |  |
| -2.649  -20.790  -7.590 | 1.2.  1.3.  2.3. |
| -31.031 |  |


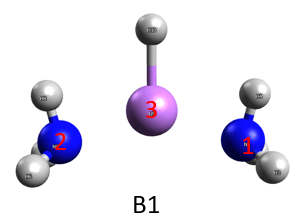


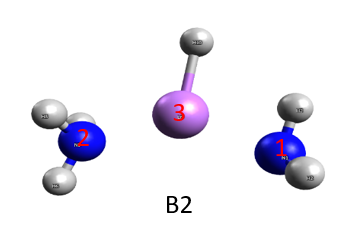

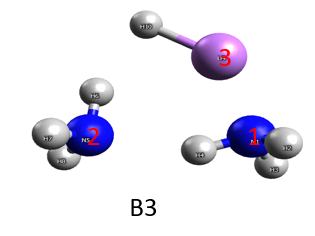


| LiH:3NH3 | | | |
| --- | --- | --- | --- |
| C1 |  |  |  |
| 2 body | fragments | 3 body | fragments |
| 2.138  2.164  2.138  -20.127  -20.120  -20.137 | 1.2.  1.3.  2.3.  1.4.  2.4.  3.4. | -0.487  0.712  0.707  0.713 | 1.2.3.  1.2.4.  1.3.4.  2.3.4. |
| -53.944 |  | 1.645 |  |
| C2 |  |  |  |
| 2 body | fragments | 3 body | fragments |
| 0.256  2.479  -2.699  -19.852  -6.345  -20.826 | 1.2.  1.3.  2.3.  1.4.  2.4.  3.4. | 0.288  -0.386  1.298  -3.624 | 1.2.3.  1.2.4.  1.3.4.  2.3.4. |
| -46.989 |  | -2.424 |  |
| C3 |  |  |  |
| 1.364  -2.912  -1.651  -20.747  -17.349  -2.618 | 1.2.  1.3.  2.3.  1.4.  2.4.  3.4. | -3.9815  1.0569  -5.6091  4.7125 | 1.2.3.  1.2.4.  1.3.4.  2.3.4. |
| -43.913 |  | 0.569 |  |
| C4 |  |  |  |
| -3.102  -2.982  -3.105  -3.374  0.838  -0.495 | 1.2.  1.3.  2.3.  1.4.  2.4.  3.4. | -1.225  0.506  -0.571  -0.046 | 1.2.3.  1.2.4.  1.3.4.  2.3.4. |
| 12.222 |  | -1.336 |  |


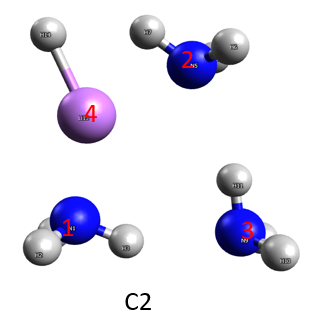

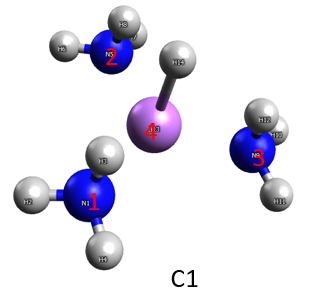

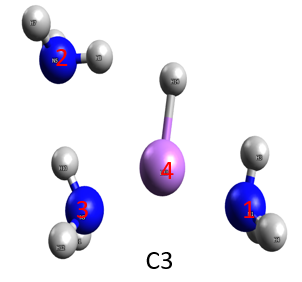

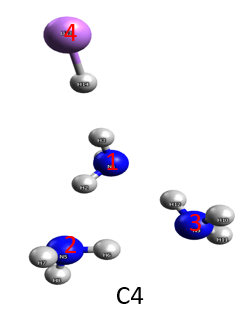


| 2 body |  | 3 body |  | 4-body |  |
| --- | --- | --- | --- | --- | --- |
| LiH:4NH3 | | | | | |
| D1 |  |  |  |  |  |
| -2.770  2.309  0.321  2.303  0.321  2.168  -20.985  -5.957  -19.860  -19.848 | 1.2.  1.3.  2.3.  1.4.  2.4.  3.4.  1.5.  2.5.  3.5.  4.5. | 0.324  0.322  -0.537  -0.067  -4.002  0.989  -0.513  0.982  -0.515  0.546 | 1.2.3.  1.2.4.  1.3.4.  2.3.4.  1.2.5.  1.3.5.  2.3.5.  1.4.5.  2.4.5.  3.4.5. | -0.037  0.350  0.351  0.197  0.210 | 1.2.3.4.  1.2.3.5.  1.2.4.5.  1.3.4.5.  2.3.4.5. |
| -61.998 |  | -2.470 |  | 1.0722 |  |
| D2 |  |  |  |  |  |
| -2.711  0.145  0.220  2.822  0.145  -2.709  -20.523  -5.933  -5.929  -20.518 | 1.2.  1.3.  2.3.  1.4.  2.4.  3.4.  1.5.  2.5.  3.5.  4.5. | 0.106  0.270  0.268  0.106  -3.865  -0.443  -0.178  1.520  -0.442  -3.866 | 1.2.3.  1.2.4.  1.3.4.  2.3.4.  1.2.5.  1.3.5.  2.3.5.  1.4.5.  2.4.5.  3.4.5. | 0.072  0.058  0.251  0.252  0.059 | 1.2.3.4.  1.2.3.5.  1.2.4.5.  1.3.4.5.  2.3.4.5. |
| -54.992 |  | -6.5225 |  | 0.693 |  |
| D3 |  |  |  |  |  |
| -2.934  -1.968  1.133  0.117  2.220  1.764  -2.263  -20.303  -16.818  -19.517 | 1.2.  1.3.  2.3.  1.4.  2.4.  3.4.  1.5.  2.5.  3.5.  4.5. | -0.459  0.059  -0.118  -0.402  -0.417  0.397  0.730  0.359  1.167  0.448 | 1.2.3.  1.2.4.  1.3.4.  2.3.4.  1.2.5.  1.3.5.  2.3.5.  1.4.5.  2.4.5.  3.4.5. | 0.057  -0.538  -0.089  -0.222  0.091 | 1.2.3.4.  1.2.3.5.  1.2.4.5.  1.3.4.5.  2.3.4.5. |
| -58.569 |  | 1.764 |  | -0.701 |  |
| D4 |  |  |  |  |  |
| 2.844  -0.336  -2.203  -2.711  1.034  -2.373  -19.829  -19.756  -5.896  -4.944 | 1.2.  1.3.  2.3.  1.4.  2.4.  3.4.  1.5.  2.5.  3.5.  4.5. | 0.114  0.089  -0.597  0.327  1.627  -0.614  -2.254  -0.835  0.617  -0.953 | 1.2.3.  1.2.4.  1.3.4.  2.3.4.  1.2.5.  1.3.5.  2.3.5.  1.4.5.  2.4.5.  3.4.5. | 0.07  0.231  -0.014  -0.193  0.124 | 1.2.3.4.  1.2.3.5.  1.2.4.5.  1.3.4.5.  2.3.4.5. |
| -54.17 |  | -2.479 |  | 0.218 |  |
| D5 |  |  |  |  |  |
| -2.717  -1.090  -2.981  1.690  0.211  -1.991  -21.120  -2.459  -0.497  -17.680 | 1.2.  1.3.  2.3.  1.4.  2.4.  3.4.  1.5.  2.5.  3.5.  4.5. | -0.785  0.027  -0.161  -0.329  -1.237  0.017  0.211  1.042  0.153  0.293 | 1.2.3.  1.2.4.  1.3.4.  2.3.4.  1.2.5.  1.3.5.  2.3.5.  1.4.5.  2.4.5.  3.4.5. | 0.002  -0.140  -0.070  -0.257  -0.120 | 1.2.3.4.  1.2.3.5.  1.2.4.5.  1.3.4.5.  2.3.4.5. |
| -48.636 |  | -0.767 |  | -0.587 |  |

**
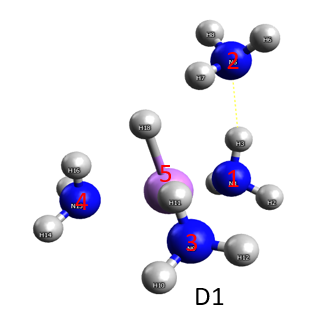

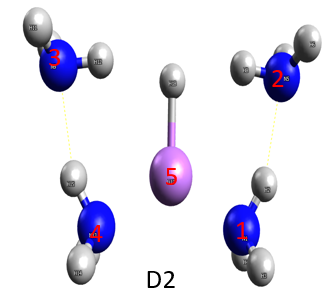

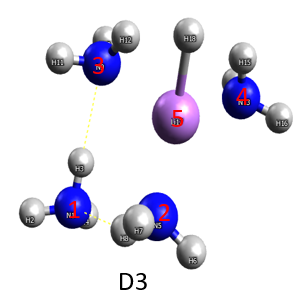

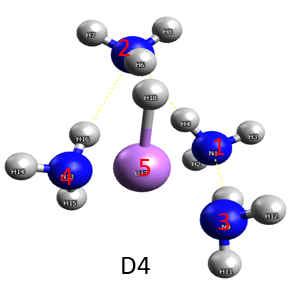

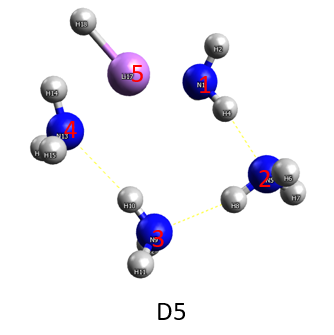
**

**Atomic coordinates of different conformer of LiH:nNH3 (n=1-4) clusters optimized at RI-MP2/ 6-311++g(2df,2pd)**

| **LiH:NH3** |
| --- |
| N 1.92494725455231 2.27010433987193 1.50664755179855  H 2.50030944296606 2.30189342310682 0.67458681082446  H 1.46796915360773 3.17097933032461 1.56961203456024  H 1.19473698720533 1.59448388210982 1.32039613239908  Li 3.00281018346513 1.81720599185475 3.18805160882980  H 3.85695899820342 1.45817939273204 4.51729152158787 |

| **Li:2NH3  (B1)** | B2 |
| --- | --- |
| N 5.138733 5.397137 4.045254  H 4.532716 6.050702 3.569037  H 5.986959 5.329981 3.500053  H 5.386651 5.810725 4.939153  N 3.517344 2.017518 5.042006  H 4.000830 1.190246 4.720905  H 2.544666 1.906948 4.791293  H 3.569487 2.022794 6.056302  Li 4.431175 3.843973 5.174697  H 4.694305 4.195670 6.795271 | N 4.97819235661218 5.38023887074549 3.97057873121298  H 5.80099506684262 5.35656276508251 3.38451692953159  H 5.24239613477950 5.81859927725344 4.84951750056421  H 4.30922019665371 5.98754541510445 3.51814972952234  N 3.64711167611310 1.98421868820163 5.13704138913099  H 3.37524269108715 1.41840833168514 4.34543047393397  H 2.83861119766552 2.06727534344975 5.74040875487828  H 4.32666246234163 1.45470004188541 5.66868342309971  Li 4.45134895215480 3.86253818312128 5.24652344932226  H 4.83308655574973 4.43560717347083 6.77311998880359 |
| B3 | **LiH:3NH3** (C1) |
| N 2.81934231007171 4.88307645555932 4.62698891501212  H 2.42755339983308 5.57873529064776 5.24708133160377  H 3.18374295155558 5.38503730958036 3.82874220953826  H 3.61794254985757 4.45434275733345 5.11134672907376  N 4.64804071524938 2.84819919795761 5.88005508878106  H 3.93121749827593 2.17402165003934 5.59020955797942  H 4.76337439584961 2.74290044484842 6.87945126900639  H 5.51899366035873 2.54906109869611 5.46155941954237  Li 1.93499426318991 3.09598761998501 4.39964688418486  H 2.27856067575840 1.53748605535251 4.79593997527780 | N 3.85086 2.50185 2.16824  H 3.04290 1.95928 1.89710  H 3.99547 2.36865 3.17027  H 4.64777 2.11099 1.68567  N 1.92901 5.28352 3.36143  H 1.03733 4.86212 3.14226  H 1.77071 6.27521 3.47189  H 2.23723 4.91351 4.26188  N 5.46816 5.61808 2.89516  H 5.46402 6.62433 2.98531  H 6.33551 5.36295 2.44425  H 5.47508 5.21958 3.83530  Li 3.76633 4.43450 2.91330  H 4.04315 3.89157 4.62491 |
| C2 | C3 |
| N 1.53091200100757 4.90145694342818 4.50119898915564  H 1.02704523556768 4.85248651259264 5.37569238140156  H 2.04478308734040 5.77839189373124 4.49296494291445  H 0.84111943591872 4.94625756097352 3.76417992541238  N 6.70218960705510 3.88414146595847 3.84976338674910  H 7.37825567880983 3.99138447610415 4.59428653901878  H 7.19338741278855 4.08637368001813 2.98911607950396  H 5.99718568900855 4.62210261345003 3.97446374094682  N 4.19015798482732 2.23512502309619 4.03932626586161  H 5.16383828242880 2.54742698994965 3.94649757039273  H 4.15353060342242 1.59501199543720 4.82103025741147  H 3.97032323405173 1.68940948486563 3.21713150114805  Li 3.41258796085082 4.10760754999670 4.23858897379564  H 4.38527403692240 5.50969552039819 4.20940553628772 | N 2.32768621416525 4.68345132381187 4.32325941029848  H 1.84430126903591 4.89999153612804 5.18462937500847  H 2.42486849664926 3.66934859247656 4.26990462468358  H 1.71158828100161 4.97199647646983 3.57522108852159  N 5.97684267986483 4.24269965442770 3.98702464478588  H 6.52775734573176 3.81390532811325 4.71989899835336  H 6.13274779303845 5.26395513478299 4.05705537962667  H 6.38364414692130 3.94762956208296 3.10842834653610  N 3.57797708859501 1.89738661656048 4.10600352347937  H 3.69322652314934 1.27078261157291 4.89033568975085  H 4.40715323285442 2.48053011662870 4.05765571211457  H 3.56279699160816 1.32218280714135 3.27538102101591  Li 4.20024559255909 5.46734304537715 4.21771439171627  H 5.21975461482548 6.81566891442608 4.24113390410877 |
| C4 | **LiH:4NH3** (D1) |
| N -1.01118506247789 2.04527302033927 0.38455467655973  H -0.71075722632162 1.99895994916638 -0.58411378437148  H -1.83619693102207 1.45810854621097 0.48036870388278  H -1.31347239804996 2.99441481399904 0.55442546276813  N 0.96166626639054 1.42764858410855 -2.15457102349590  H 1.42833115983700 1.14629988557600 -1.29495138857491  H 0.86569561845138 0.60156231264581 -2.72808168769321  H 1.58787246702978 2.04857204135844 -2.64737385928627  N 1.78720243010082 0.80975235643365 0.85912137040265  H 1.75462764427642 -0.09933218173701 1.29885474112646  H 2.42081762125205 1.37164081338345 1.41021343997410  H 0.85721432116649 1.22299747000996 0.94592709311921  Li -4.65667102966921 -0.66683883937716 0.88563259986198  H -3.43658488096374 0.33594122788251 0.73308365572674 | N 5.01128179867519 3.04945927443844 6.60510623323747  H 4.97893675740569 3.80184727427094 7.27946681430579  H 5.92798220680828 3.08834248538929 6.14876458545542  H 4.97632016253701 2.19047312703241 7.13691119247660  N 7.28286993015889 3.22255992353122 4.60705560807113  H 7.83741909853874 4.04942899789135 4.42918741924918  H 6.45500666115651 3.27772109094076 3.99877933304056  H 7.83479111300592 2.43796764873493 4.28663053116310  N 2.83930822030289 4.99582211640669 4.49996710798561  H 1.89027583358683 5.06366618336408 4.16024989007661  H 3.45352725096106 4.84952487610505 3.69632702942437  H 3.07403508626747 5.88957134117636 4.90844795794878  N 2.83369021240817 1.50991535725932 4.19154290785754  H 1.88454056528937 1.50580824481722 3.84546316078128  H 3.06544395551892 0.55758282518340 4.43668352248201  H 3.44859826146769 1.79308067195599 3.42589880944961  Li 3.83584551380088 3.20005139208726 4.92450855773276  H 4.75143606211038 3.33884775941517 3.33910974926203 |
| D2 | D3 |
| N -1.51360856643413 3.40821237336909 -1.90731920108649  H -1.98012044249795 4.29519641790407 -1.68862570290647  H -0.51772392095349 3.58196013589549 -1.88900910886980  H -1.74127661295206 3.17948518190839 -2.86553649171443  N -3.20699055131129 5.59151050392557 -0.69381290769350  H -2.85966731083839 6.32902261752098 -0.09548380464136  H -4.08328508535766 5.92251999543469 -1.07472069588677  H -3.42355856651309 4.78863019714685 -0.09048721278353  N -3.95556255776152 0.77884618213340 2.20453379057665  H -4.92579433897399 0.49426759828900 2.18956696517707  H -3.70828022071990 0.89697272207757 3.17789113477240  H -3.90526861995092 1.70581486226973 1.76441012424460  N -1.99191028348660 0.31247249407567 -0.03926510908798  H -1.06703226895773 0.03183159178827 0.25769225849652  H -2.61288987280151 0.21477787533929 0.77133884165958  H -2.28582272758007 -0.37035444224485 -0.72461624482335  Li -2.35822654880448 2.30021812191190 -0.39955949426027  H -3.46617150410427 3.10245557125391 0.64787285882716 | N 5.53979600771641 3.49935616754551 7.35695391902995  H 5.84512754552357 4.26056802398156 7.94687614434872  H 4.74445209569557 3.82816654340439 6.81680206256228  H 5.20948223031349 2.76862174245848 7.97145334427752  N 6.34654066235206 2.92140847054337 4.44229610821182  H 6.76677313666965 2.03181471555838 4.21187526561835  H 6.91742981090578 3.63196246518421 4.00496229044263  H 6.41668853177512 3.04294931101276 5.45153436868482  N 3.31192065204002 4.66872853611104 5.24957352561763  H 2.39318909463724 4.50890934490585 5.64344571625460  H 3.59798186255532 5.59013268392266 5.55696694611885  H 3.19011688956964 4.73338205179569 4.21910629683558  N 3.07207740249253 1.56797168008678 3.71506545505052  H 2.26769313571063 1.35765194349948 4.28886090952613  H 2.78964724613037 2.25091234957865 3.00842181889383  H 3.32650419988576 0.71718353569671 3.23297894571080  Li 4.37379631224202 3.18249289366351 3.97138425415656  H 3.57916979378464 4.08821823105085 2.65969187865923 |
| D4 | D5 |
| N 4.96479234314261 2.98313112646614 7.36228147087271  H 4.58673402080497 2.26911099558967 6.73571777495497  H 5.74635475235066 2.57580797094439 7.85537607083725  H 4.25336209612736 3.18882004632912 8.05022461949568  N 6.17795985903510 4.02716233269509 4.31420396626141  H 5.39099565462132 3.70775666904997 3.75331168833693  H 6.47754438880494 4.90236006623390 3.90508668892692  H 6.93343111760637 3.36715097881009 4.19543058177077  N 3.22569408118916 3.93655890385408 3.23003066407374  H 2.25696714821539 3.65150628695942 3.15010194369676  H 3.27841734251067 4.57087218348908 4.04792830975241  H 3.40182518185805 4.50935720771460 2.41330576694225  N 3.72397015967470 1.35065281035264 5.04366752600741  H 4.13601872084125 0.55580691150435 4.57393765020358  H 2.78348008506763 1.07471975955139 5.29321533086893  H 3.63386759697844 2.09176453582112 4.35032590041756  Li 5.01728795139496 4.45799503627798 5.96604383931898  H 3.69968409977571 5.42989688835641 5.50805942726081 | N 1.03551274771387 1.04852216087845 2.02772730031465  H 1.00021374824239 0.16577310071500 2.52193309813921  H 0.07356895679284 1.29043425877052 1.82888310706489  H 1.38725838602187 1.74935591959776 2.68594986499918  N 2.28330537142600 3.28437867777299 3.71184347577530  H 3.15162474282498 3.07883996274176 4.18648125599988  H 1.74946537676770 3.87473814090346 4.33445096350754  H 2.50883083887588 3.83570598123715 2.88527236380124  N 2.70209016613084 4.56082767086165 0.85754701278950  H 2.74308336613744 3.70843140196391 0.30821604313099  H 3.52898493348266 5.09804102418192 0.63548034851325  H 1.91404492353748 5.09457531082698 0.51742432525115  N 3.08290692546841 1.90128269528080 -1.11731281409113  H 3.07704934736610 0.88324449128065 -1.31125884200240  H 4.05247790487829 2.19168612188290 -1.13482101077381  H 2.62846807076518 2.35541564483728 -1.89925083939048  Li 2.13171082182602 0.67898849097929 0.36949110319403  H 2.54766582174177 -0.66745901471278 -0.58163374622308 |
